# SUV39H1 regulates the progression of *MLL*-*AF9*-induced acute myeloid leukemia

**DOI:** 10.1038/s41388-020-01495-6

**Published:** 2020-10-09

**Authors:** Yajing Chu, Yangpeng Chen, Huidong Guo, Mengke Li, Bichen Wang, Deyang Shi, Xuelian Cheng, Jinxia Guan, Xiaomin Wang, Chenghai Xue, Tao Cheng, Jun Shi, Weiping Yuan

**Affiliations:** 1 State Key Laboratory of Experimental Hematology, National Clinical Research Center for Hematological Diseases, Institute of Hematology and Blood Diseases Hospital, Chinese Academy of Medical Sciences and Peking Union Medical College, 300020 Tianjin, China; 2 My Health Gene Technology Co., Ltd., Service Centre of Tianjin Chentang Science and Technology Commercial District, 300220 Tianjin, China; 3 Present Address: Beijing Key Laboratory of Hematopoietic Stem Cell Transplantation, National Clinical Research Center for Hematologic Diseases, Peking University People’s Hospital, Peking University Institute of Hematology, 100044 Beijing, China

**Keywords:** Acute myeloid leukaemia, Gene silencing, Cancer stem cells

## Abstract

Epigenetic regulations play crucial roles in leukemogenesis and leukemia progression. SUV39H1 is the dominant H3K9 methyltransferase in the hematopoietic system, and its expression declines with aging. However, the role of SUV39H1 via its-mediated repressive modification H3K9me3 in leukemogenesis/leukemia progression remains to be explored. We found that *SUV39H1* was down-regulated in a variety of leukemias, including *MLL*-r AML, as compared with normal individuals. Decreased levels of *Suv39h1* expression and genomic H3K9me3 occupancy were observed in LSCs from *MLL*-r-induced AML mouse models in comparison with that of hematopoietic stem/progenitor cells. *Suv39h1* overexpression increased leukemia latency and decreased the frequency of LSCs in *MLL*-*r* AML mouse models, while *Suv39h1* knockdown accelerated disease progression with increased number of LSCs. Increased *Suv39h1* expression led to the inactivation of *Hoxb13* and *Six1*, as well as reversion of Hoxa9/Meis1 downstream target genes, which in turn decelerated leukemia progression. Interestingly, *Hoxb13* expression is up-regulated in *MLL*-*AF9*-induced AML cells, while knockdown of *Hoxb13* in *MLL*-*AF9* leukemic cells significantly prolonged the survival of leukemic mice with reduced LSC frequencies. Our data revealed that SUV39H1 functions as a tumor suppressor in *MLL*-*AF9*-induced AML progression. These findings provide the direct link of SUV39H1 to AML development and progression.

## Introduction

Acute myeloid leukemia (AML) is the most frequent type of acute leukemia in adults. However, there have been few significant advances in the past 40 years beyond the use of conventional chemotherapy in combination with bone marrow (BM) transplantation for AML treatment [1, 2]. Therefore, investigating the underlying mechanisms of AML initiation and progression is needed to find novel treating targets/strategies for AML.

Genome-wide analyses have shown that genes involved in epigenetic modifications are the most recurrently mutated genes in AML, suggesting crucial roles of epigenetic regulations in AML leukemogenesis and progression [3, 4]. These genes, including the methylcytosine hydroxylase TET2, DNA methyltransferase DNMT3A, and Polycomb-related protein ASXL1, directly contribute to the aberrant distribution of epigenetic modifications in AML [5].

Leukemia bearing translocations involving chromosome 11q23 is one of the most aggressive and drug-resistant leukemias, characterized by the presence of Mixed Lineage Leukemia (*MLL*) rearrangements. More than 79 fusion genes have been reported to translocate with *MLL*, sharing some correlation with disease phenotype and prognosis [6–8]. *MLL*-r leukemia exhibits a distinct characteristic of pathology and molecular mechanism from other subtypes of AML. It has been reported that *MLL*-r leukemia stem cells (LSCs) exhibits aberrantly increased levels of H3K4me3 and H3K79me2 [9–13]. The high level of H3K4me3 in LSC, due to the decreased expression of H3K4-specific demethylase KDM5B [12], was shown to be associated with LSC maintenance. In addition, the hyperactive H3K79 methyltransferase DOT1L has been reported to be responsible for the aberrant H3K79me2 in *MLL*-r leukemia [11, 13]. It was reported that the high levels of lysine-specific demethylase KDM1A (catalyzes the demethylation of H3K4me1/2 and H3K9me1/2) was essential for *MLL*-r induced leukemia [14]. SUV39H1, the first characterized mammalian lysine methyltransferase, catalyzes the di- and tri-methylation of histone 3 lysine 9 [15]. SUV39H1 is the predominant H3K9 methyltransferase expressed in mouse long-term hematopoietic stem cells (LT-HSCs), human cord blood cells and BM CD34^+^CD38^–^ HSCs [16]. Report showed that SUV39H1 expression declines with aging in human and mouse HSCs, leading to a global H3K9me3 reduction and perturbed heterochromatin function [16]. These results suggest a tight connection of SUV39H1 with aging, which is considered as one of the strongest predisposition factors for myeloid malignancy [17]. Interestingly, SUV39H1 has been reported to interact with Menin [18], which interacts with *MLL*-fusion proteins to promote oncogenesis [19, 20]. In *MLL*-r leukemic cells, Dot1L, via its binding to the DNA, interrupts the normal localization of Suv39h1 and Sirt1, and deletion of *Suv39h1* desensitizes leukemic cells to Dot1L inhibition [21]. In addition, Dot1L-mediated H3K79 methylation requires the predisposition of H3K9 acetylation, which is recognized by AF9 [22]. These studies suggest that SUV39H1 plays a role in *MLL*-r AML.

Here we examined the expression patterns of *SUV39H1* in *MLL*-r AML and normal hematopoietic cell populations, and its correlation with AML outcomes. We further dissected the role of SUV39H1 in *MLL*-r induced AML progression, and explored the underlying molecular pathways mediated by SUV39H1 in *MLL*-r AML progression. Our studies revealed a tumor suppressive role of Suv39h1 in *MLL*-r AML that is partially mediated by inactivation of *Hoxb13* and *Six1*, as well as reversion of Hoxa9/Meis1 downstream transcriptomes.

## Results

### Decreased expression of *SUV39H1* in both human and mouse AML leukemia stem cells in comparison with their normal counterparts

**Fig. 1:**
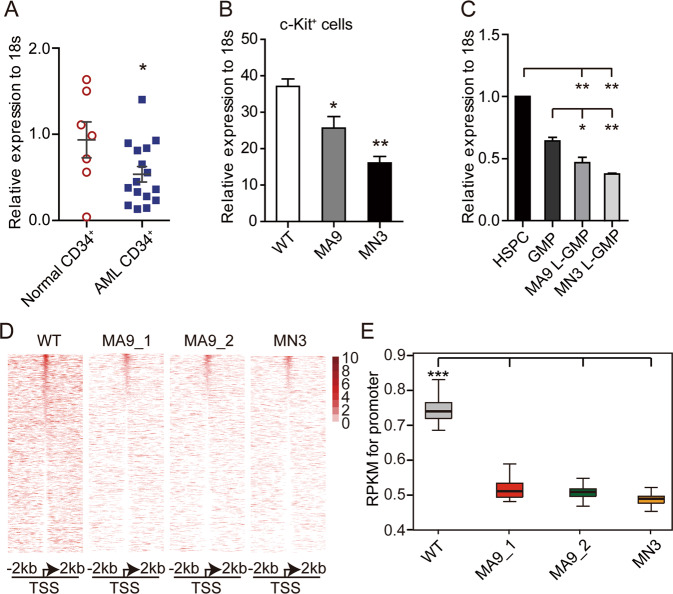
Differential expression of *SUV39H1* and distribution of H3K9me3 in *MLL*-r AML LSCs in comparison with normal counterparts. **a** Expression levels of *SUV39H1* in our in-house collected samples: bone marrow (BM) CD34^+^ cells from donors and AML patients. Data are presented as means ± s.e.m., *P < 0.05, Student’s *t* test. **b**, **c** Expression levels of *Suv39h1* in leukemic stem cell-enriched groups (defined as c-Kit^+^ in **b**, and as L-GMP, IL-7R^–^Lin^–^Sca-1^–^c-Kit^+^CD34^+^CD16/32^+^ in **c**) isolated from two *MLL*-r AML mouse models compared with the expression in normal murine HSPCs. MA9, MLL-AF9; MN3, MLL-NRIP3 (see Materials and Methods). Data are presented as means ± s.e.m., *n* = 3, *P < 0.05, **P < 0.01, Student’s *t* test. **d** Heat maps showing ChIP-seq signal of H3K9me3 at TSSs ± 2 kb regions for all genes in c-Kit^+^ cells isolated from WT or *MLL*-r leukemic mice. **e** Box plots showing changes in ChIP-seq signals of H3K9me3 at TSSs ± 2 kb regions of genome in c-Kit^+^ cells isolated from WT or MLL-r leukemic mice. ***P < 0.001, Wilcoxon test.

To examine the potential clinical relevance of SUV39H1 to human AML, the expression pattern of *SUV39H1* was assessed in two public AML databases in BloodSpot [23]. *SUV39H1* expression was significantly lower in a variety of leukemias, including the *MLL*-r AML, when compared with its expression in granulocyte-monocyte progenitor cells (GMPs) (Supplementary Fig. 1a) or with the nearest normal counterpart (Supplementary Fig. 1b), as recently published study [24]. In addition, the expression level of *SUV39H1* in stem/progenitor-enriched CD34^+^ AML cells was significantly lower than in normal cord blood CD34^+^ cells, which may either be due to the age effect [16] or the combinative effects of age and leukemia (Fig. 1a). We further explored the correlation between the expression level of *SUV39H1* and patient survival data using PrognoScan [25] and found that there was a trend that higher level of *SUV39H1* predicted better prognosis when patients were separated according to the median expression (Supplementary Fig. 1c–e). Using MLL-AF9 (MA9)-induced and MLL-NRIP3 (MN3)-induced AML mouse models, we further examined the expression level of *Suv39h1* in murine LSCs in comparison with HSPCs. We found that *Suv39h1* expression was reduced in both c-Kit^+^ defined LSCs [26] and L-GMP (labeled as Lin^−^Sca1^−^IL-7R^−^c-Kit^+^CD34^+^FcR-rII/III^+^) LSCs [27], as compared with their normal counterparts (Fig. 1b, c). These data suggest a potential role of Suv39h1 in AML maintenance.

### Differential distributions of H3K9me3 in normal hematopoietic stem/progenitor cells and leukemia stem cells

Suv39h1 has been shown to be the most abundant H3K9 methyltransferase in HSPCs [16, 17]. This observation was confirmed by using droplet digital PCR for absolute quantification of H3K9 methyltransferases in c-Kit^+^ cells (Supplementary Fig. 1f). Since the aberrant distribution of H3K9me3 was observed widely in AML patients [28], we therefore, hypothesized that decreased expression of Suv39h1 may contribute to a reduction of genomic H3K9me3 occupancy in LSCs. To gain an overview of H3K9me3 occupancy in *MLL*-r AML, we performed H3K9me3 chromatin immunoprecipitation sequencing (ChIP-seq) in c-Kit^+^ cells from WT controls, MA9 and MN3 AML mice. The distributions of H3K9me3 on the whole genome and around TSS (transcriptional start site, defined as TSS ± 2 kb) were significantly lower in AML LSCs than in normal HSPCs (Fig. 1d, e and Supplementary Fig. 2a, b). Consistent with previous H3K9me3 ChIP-seq data in human peripheral blood mononuclear cells from ENCODE [GSM613878], the H3K9me3 peaks in the WT, MA9, and MN3 groups had a higher chance of being located in intergenic and intron regions (Supplementary Fig. 2c). Gene ontology (GO) analyses of the H3K9me3 differentially enriched genes revealed an involvement of several important signaling pathways mis-regulated in cancers (Supplementary Fig. 2d), suggesting a dysregulated distribution of H3K9me3 in leukemogenesis.

### *SUV39H1* expression levels affect the leukemic progression of *MLL-AF9-*induced AML

**Fig. 2:**
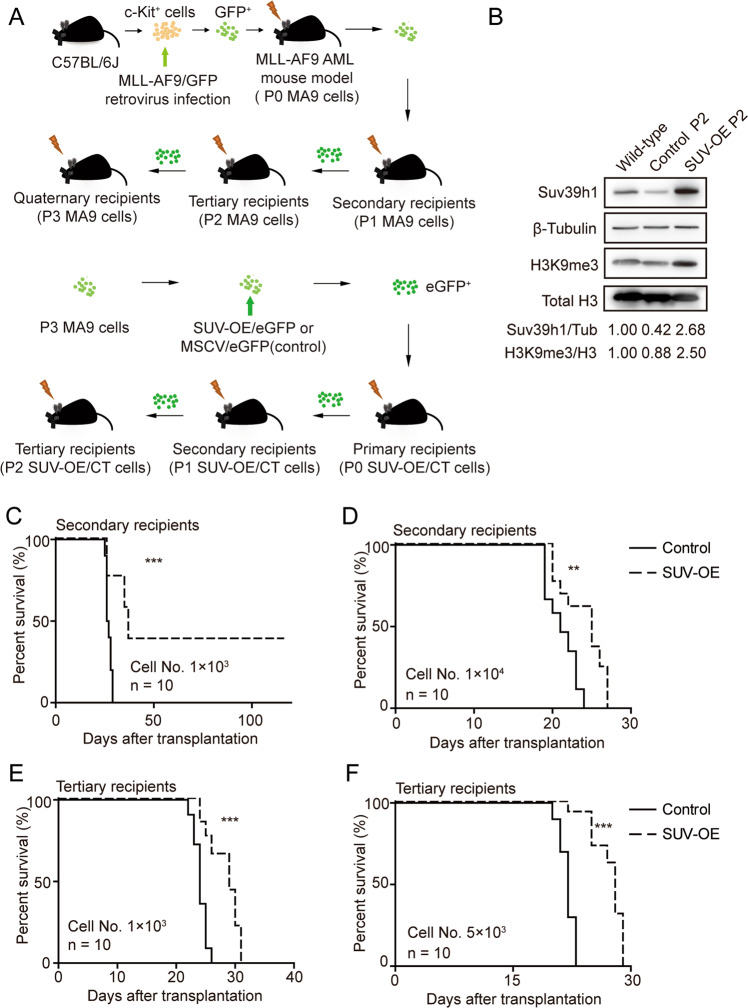
Restoring *SUV39H1* expression suppressed leukemic progression of *MLL-AF9*-induced murine AML. **a** Experimental scheme of establishing Suv39h1 overexpressing (SUV-OE) and control murine AML models. **b** Immunoblot analysis of Suv39h1 and H3K9me3 levels in whole BM cells from control or SUV-OE tertiary recipients. Densitometry was determined by ImageJ, *n* = 2. **c**, **d** Kaplan–Meier survival curve of secondary recipients (transplanted with P0 cells, 1 × 10^3^ cells per group for (**c**) and 1 × 10^4^ cells per group for (**d**). *n* = 10, **P < 0.01, ***P < 0.001, Mantel–Cox test. **e**, **f** Kaplan–Meier survival curve of tertiary recipients (transplanted with P1 cells, 1 × 10^3^ cells per group for (**e**) and 5 × 10^3^ cells per group for (**f**). *n* = 10, ***P < 0.001, Mantel–Cox test.

**Fig. 3:**
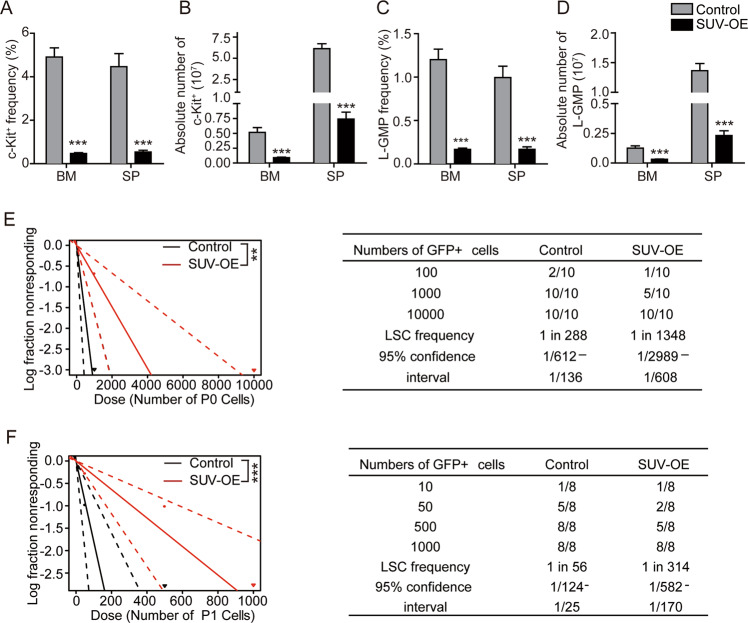
*SUV39H1* overexpression reduced the frequency of leukemia stem cells in *MLL-AF9*-induced murine AML. **a**, **c** Cell frequency analyses of c-Kit^+^ (**a**) and L-GMP (**c**) in bone marrow (BM) and spleen (SP) from moribund tertiary recipients using flow cytometry. *n* = 6, ***P < 0.001, Student’s *t* test. **b**, **d** Absolute cell number of c-Kit^+^ (**b**) and L-GMP (**d**) in BM and SP from moribund tertiary recipients. Leukemic cells were collected from femur, tibia and ilium (for BM) and whole SP (for SP), and dead cells were excluded using Trypan blue staining. *n* = 6, ***P < 0.001, Student’s *t* test. **e**, **f** Limiting dilution assay estimation of LSC frequency of P0 (**e**) and P1 (**f**) AML cells. Left panels: Logarithmic plot showing the percentage of non-responding recipients transplanted with different cell doses of eGFP^+^ P0 (**e**) and P1 (**f**) BM cells. Right panels: Table showing the number of recipients that developed leukemia and total number of recipients transplanted per cell dose. The chi-squared test was used. **P < 0.01, ***P < 0.001.

To delineate the role of SUV39H1 in *MLL*-r AML, we used a lentivirus-mediated transduction system to restore the expression of *Suv39h1* in MA9 BM AML cells. MA9 leukemia cells isolated from quaternary recipients (P3 MA9 cells) that were infected with Suv39h1-overexpression lentivirus have been stably passaged for three times (Fig. 2a). Sorted eGFP^+^ cells were transplanted to sublethally-irradiated recipient mice (Supplementary Fig. 3a). Western blotting analysis confirmed the restored expression of Suv39h1 (about 1.7-fold increase than WT cells) with a moderate increase in global H3K9me3 levels in Suv39h1-overexpressed (SUV-OE) P2 cells (Fig. 2b). Consistent with qRT-PCR result (Fig. 1b), we observed a decrease of Suv39h1 in regular AML cells in comparison with normal BM cells (Fig. 2b). The stably passaged SUV OE MLL-AF9 leukemia cells are highly enriched with LSCs and rather malignant. Once transplanted, mice can quickly develop leukemia and died in a short period of time. To ensure that the survival experiment data of leukemic mice are more discernable and accurate, we chose two relatively low doses of leukemia cells (Cell No. 1 × 10^3^ or 1 × 10^4^) for transplantation in our study. Significantly, Suv39h1 overexpression prolonged the survival of secondary murine recipients (Fig. 2c, d), as well as in the tertiary recipients (Fig. 2e, f). Moreover, immunoblot analyses of SUV-OE MLL-AF9 leukemia cells (P1 and P2 cells) showed that overexpression of Suv39h1 increased H3K9 trimethylation level in the whole BM cells isolated from secondary and tertiary recipient mice (Supplementary Fig. 3b, c). Considering the crucial role of LSC in the initiation and maintenance of leukemia, we further analyzed the effect of Suv39h1 on LSCs in MA9 AML mouse model. As assessed by immunophenotype, the frequency and absolute number of c-Kit^+^ and L-GMP LSCs in BM and SP were significantly decreased in SUV-OE groups when compared with those in controls (Fig. 3a–d and Supplementary Fig. 3d). Functional analyses with limiting dilution assays revealed an approximately fivefold and sixfold decreases in LSC numbers in SUV-OE AML cells from primary and secondary recipients, respectively (Fig. 3e, f).

### Restored expression of Suv39h1 induced apoptosis and suppressed proliferation of LSCs

**Fig. 4:**
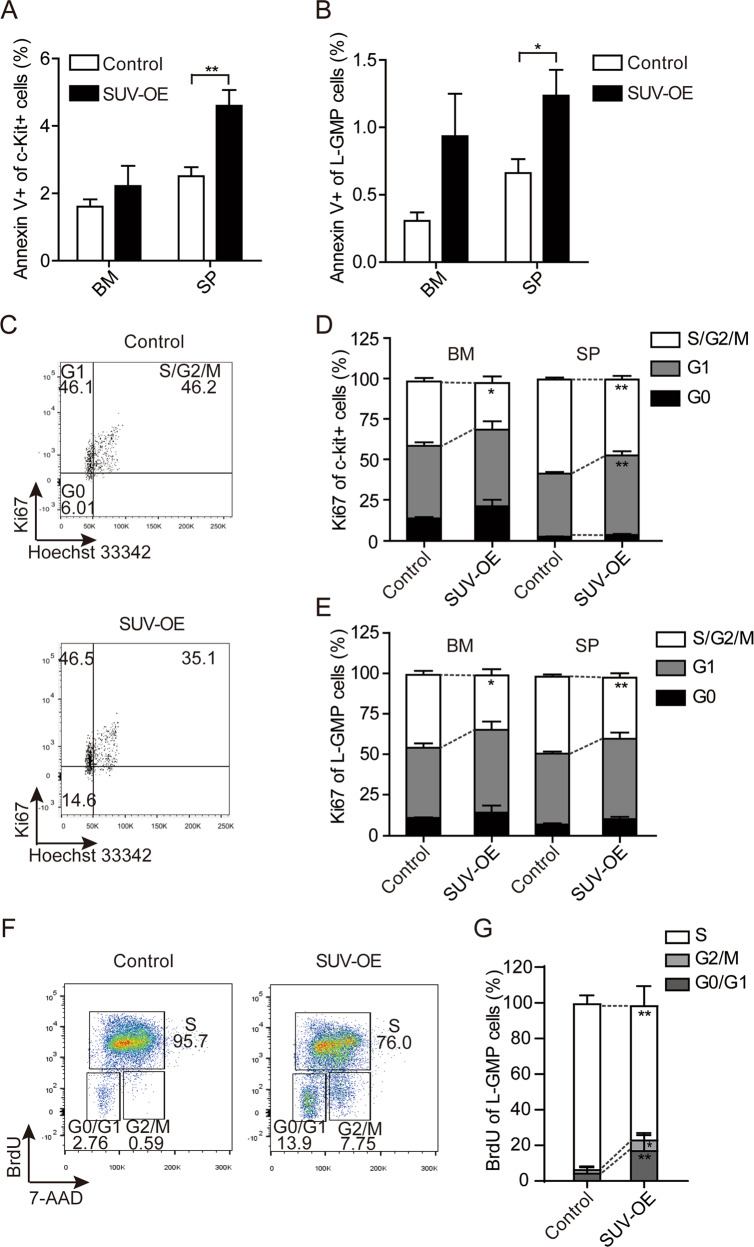
Increase expression of *Suv39h1* induced apoptosis and suppressed proliferation of leukemia cells. **a**, **b** Apoptosis analyses of c-Kit^+^ (**a**) and L-GMP (**b**) LSCs in BM and SP from moribund tertiary recipients. *n* = 7 for each group. *P < 0.05, **P < 0.01, Student’s *t* test. **c** Representative flow cytometric analysis of cell cycle of L-GMP in BM from tertiary recipients. **d**, **e** Quantification of G0, G1 and S/G2/M phases of c-Kit^+^ (**d**) and L-GMP (**e**) cells in BM and SP from tertiary recipients. *n* = 7 for each group. *P < 0.05, **P < 0.01, ***P < 0.001, Student’s *t* test. **f** Flow cytometry was used to evaluate proliferation of leukemia stem cells (L-GMP) in SUV-OE (*n* = 8) and Control (*n* = 9) mice (BrdU incorporation assay). **g** Quantification of G0/G1, S and G2/M phases of L-GMP cells in BM (**f**). *P < 0.05, **P < 0.01, Student’s *t* test.

The reduced frequency of LSCs by SUV-OE in MA9 AML mice prompted us to further investigate whether SUV-OE alters the apoptosis and cell cycle in LSCs. We observed a slightly increased percentage of apoptotic c-Kit^+^ and L-GMP LSCs in BM and SP in the SUV-OE mice group (Fig. 4a, b). Flow cytometry analysis of c-Kit^+^ LSCs from BM and SP revealed a significantly decreased proportion of cells at the S/G2/M phase and a concordant increased proportion of G0/G1 phases in SUV-OE c-Kit^+^ LSCs cells when compared with the proportions in control cells (Fig. 4c, d). Further analyses of the cell cycle status with either Ki67 staining or BrdU incorporation of L-GMP cells revealed similar results (Fig. 4e–g). Interestingly, since Ki67 is not expressed in G0 phase, we could distinguish that the increased G0/G1 phase is mainly due to the increased of G1 phase but not G0 phase. These data collectively showed that restoration of Suv39h1 expression level induces the apoptosis of LSCs and impairs the proliferation of LSCs.

To substantiate our in vivo observations in *MLL*-r murine model, we further restored the expression of *SUV39H1* in human THP-1, Molm13 and Nomo-1 cell lines, which bearing the *MLL* fusion gene (Supplementary Fig. 4a–c). Colorimetric-based CCK8 assay showed that restoration of SUV39H1 expression significantly suppressed the cell growth in these three cell lines (Supplementary Fig. 4d–f) and the proliferation abilities of Nomo-1 cells (Supplementary Fig. 4g–j). Furthermore, the apoptotic ratios of SUV-OE cell lines were much higher than that of the controls (Supplementary Fig. 4k–p). In addition, Suv39h1 shRNA knockdown (Supplementary Fig. 4q, r) could promote the cell growth of THP-1 and Molm13 cells (Supplementary Fig. 4s, t) and cell cycling of Molm13 cells (Supplementary Fig. 4u, v) when compared with the controls. Collectively, these results suggest a tumor suppressive role of SUV39H1 in both *MLL*-r AML mouse model and human cell line.

### Suppression of *Suv39h1* accelerated the leukemic progression in *MLL-AF9-*induced AML

**Fig. 5:**
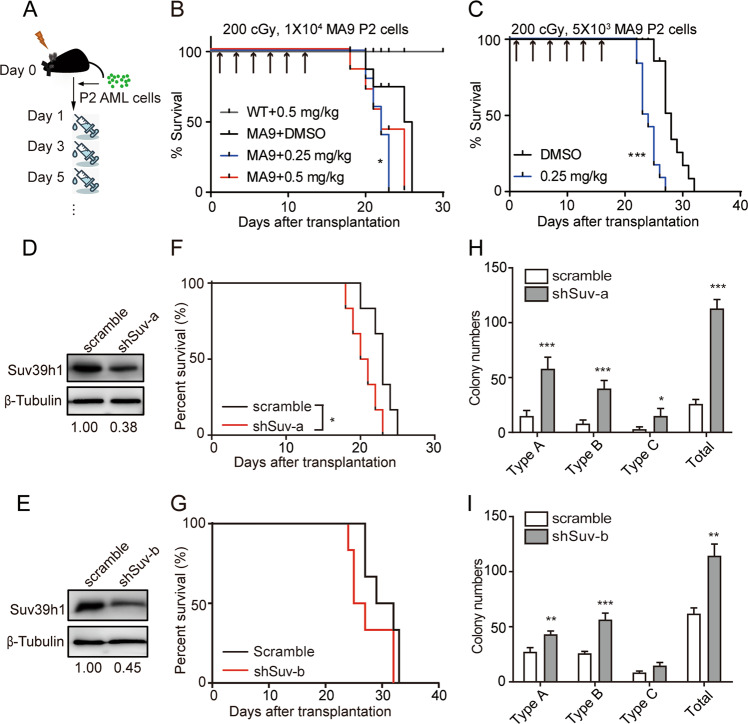
Chaetocin treatment or *Suv39h1* knockdown accelerated AML progression. **a** Experimental scheme of chaetocin treatment. Mice were sub-lethally irradiated, and transplanted with MA9 P2 AML cells. Chaetocin treatment started at 1 day after transplantation. Intraperitoneal (ip) injections were done every 2 days. **b** Kaplan–Meier survival curve. Mice were treated as described in (**a**). Median survival: 25.5 days for DMSO group, 22 days for both dosages of chaetocin groups. *n* = 5 for each group. Mantel–Cox test, *P < 0.05 for 0.25 mg/kg chaetocin group only. **c** Kaplan–Meier survival curve. Mice were treated as described in (**a**). Median survival: 28 days for DMSO group, 23.5 days for chaetocin group. *n* = 12 for each group. Mantel–Cox test, ***P < 0.0001. **d**, **e** Immunoblot analyses of Suv39h1 level in whole BM cells from control or shSUV primary recipients (**d** for shSuv-a and **e** for shSuv-b). Densitometry was determined by ImageJ. **f**, **g** Kaplan–Meier survival curves of secondary (transplanted with P0 cells, 2 × 10^4^ cells per group, (**f**) for shSuv-a and (**g**) for shSuv-b) recipients. *n* = 6 for each group, Mantel–Cox test *P < 0.05 for shSuv-a. **h**, **i** Colony-forming assays of BM leukemic cells from secondary recipients (**h** for shSuv-a and **i** for shSuv-b); *n* = 4, *P < 0.05, ***P < 0.001, Student’s *t* test.

Chaetocin was the first reported inhibitor for SUV39H1/H2 [29], although the follow-up studies showed that this inhibition was not totally specific and time-dependent [30–32]. Recent study indicates a suppressive role of chaetocin in human AML cell lines and in a xenograft mouse model (none of these cell lines bearing *MLL*-*r* translocation as reported) [33, 34]. Since there isn’t a specific inhibitor of SUV39H1, we therefore used chaetocin to test the potential effect of this non-specific inhibitor of SUV39H1 in MA9 AML mouse models. We examined the effect of chaetocin treatment with different dosages and regimens during leukemia progression. First, sub-lethally irradiated mice were transplanted with 1 × 10^4^ MA9 P2 AML cells, then treated with chaetocin or vehicle one day after transplantation (Fig. 5a). Shortened survivals were observed in low dosage (0.25 mg chaetocin /kg) group. A slightly accelerated disease progression was also observed in high dosage group (0.5 mg/kg, the same dosage as used in ref. [34]) (Fig. 5b). No obvious body weight changes were observed in low dosage group in comparison with vehicle group (Supplementary Fig. 5a). The same treatment but with lower input leukemia cells also showed accelerated disease progression when treated with chaetocin (Fig. 5c and Supplementary Fig. 5b). If chaetocin treatment started around 5% GFP^+^ cells in peripheral blood, no significant differences were observed between chaetocin and vehicle treatment group (Supplementary Fig. 5c–h). We confirmed that the level of H3K9me3 was decreased upon chaetocin (higher dose) treatment (Supplementary Fig. 5i, j).

Considering the non-direct effect of chaetocin on SUV39H1 [30–32], we further explored whether direct suppression of *Suv39h1* would accelerate leukemia progression by reducing the expression of *Suv39h1* using shRNA-mediated knockdown (Fig. 5d, e). *Suv39h1* knockdown with shSUV-a accelerated the progression of leukemia with an average of 20.5-day survival in shSUV-a group vs. 23-day in scramble established in parallel with shSUV-a (Fig. 5f), and 26-day survival in shSUV-b group vs. 30.5-day in its parallel scramble (Fig. 5g). The effect of *Suv39h1* knockdown on survival was further reconfirmed with simultaneously established scramble, shSuv-a, and shSuv-b groups. An average of 24-day survival in shSUV-a, 25-day survival in shSUV-b respectively vs. 28-day in scramble group were observed (Supplementary Fig. 6a, b). The levels of H3K9me3 were decreased accompanied with *Suv39h1* knockdown (Supplementary Fig. 6c, d). In addition, the LSC frequencies measured by the colony-forming capacity assay, were increased in sh-SUV groups as compared with the controls (Fig. 5h, i). These data demonstrated that *Suv39h1* is a critical regulator of leukemia progression in *MLL*-r AML.

### Suv39h1 overexpression led to dysregulation of transcriptional program involved in leukemia

**Fig. 6:**
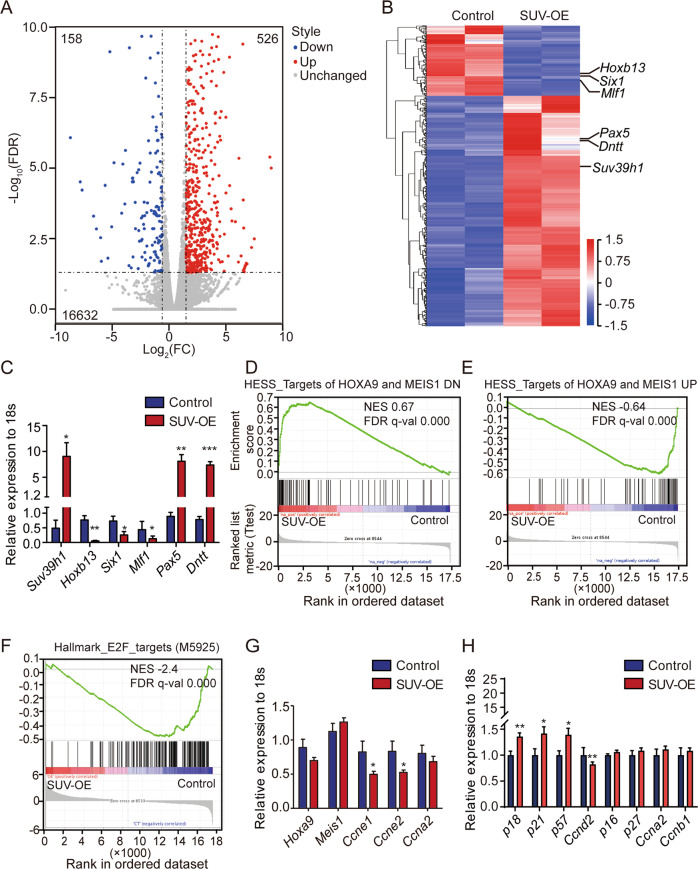
*Suv39h1* overexpression resulted in dysregulation of transcriptional program involved in leukemia. **a** Control and SUV-OE P1 AML c-Kit^+^ cells from moribund mice were used for RNAseq analysis. Volcano plot of the transcriptome profile is presented. Red dots and blue dots represent differentially expressed genes (DEGs) with a log_2_FC ≥ 1 and FDR < 0.05 (DESeq2) or log_2_FC ≤ −1 and FDR < 0.05, respectively, and black dots represent genes with no significant differences between control and SUV-OE. *n* = 2 per group. **b** Heatmap showing the DEGs, |log_2_FC| ≥ 1, FDR < 0.05 between control and SUV-OE AML c-Kit^+^ cells using RNAseq analysis. **c**, **g** Quantitative RT-PCR analysis of DEGs revealed using RNA-Seq in control and SUV-OE c-Kit^+^ cells. Data are presented as the means ± s.e.m., *n* = 3, *P < 0.05, **P < 0.01, ***P < 0.001, Student’s *t* test. **d**, **e** Gene set enrichment analysis demonstrating increased gene expression of the down-regulated targets and decreased expression of upregulated targets of HOXA9 and MEIS1 in SUV-OE groups compared with the expression in controls. NES (nominal enrichment score), FDR (false discovery rate). **f** Gene set enrichment analysis showing decreased expression of hallmark of E2F targets in SUV-OE groups compared with the expression in controls. **h** qRT-PCR analysis of cell cycle related genes. Data are presented as the means ± s.e.m., *n* = 3, *P < 0.05, **P < 0.01, ***P < 0.001, Student’s *t* test.

To examine the underlying mechanisms by which Suv39h1 restoration suppresses *MLL*-r leukemia progression, gene expression profiles were assessed using RNA-Seq in LSCs. A total of 684 genes were differentially expressed (|log_2_FC| ≥ 1 and FDR < 0.05). Among these genes, 158 were down-regulated, and 526 were upregulated (Fig. 6a, b). KEGG pathway analyses of these differentially expressed genes (DEG) showed enrichment of transcriptional mis-regulation in cancer, ECM-receptor interaction, hematopoietic cell lineage, and several signaling pathways, such as PI3K-Akt pathway, FOXO pathway and p53 pathway (Supplementary Fig. 7a). Most of these pathways were consistent with the differential H3K9me3 peaks in LSCs vs. HSCs (Supplementary Fig. 2d). The relationships between these enriched KEGG pathways of DEGs were further dissected with Cytoscape. These KEGG pathways primarily fell into two groups: one group contained up-regulated apoptosis as one of the most central pathways, and the other contained down-regulated metabolic pathways (Supplementary Fig. 7b). The upregulated apoptosis was consistent with the increased apoptosis phenotype in SUV-OE LSCs from MA9 AML mice (Fig. 4a, b). The dysregulated metabolic pathways might be due to the decreased expression of transcription factor *Six1* (Fig. 6b, c), which was reported as a regulator of metabolism [35, 36].

Gene set enrichment analysis [37] demonstrated an increased expression of suppressed target genes by HOXA9 and MEIS1, and a decreased expression of the activated target genes by HOXA9 and MEIS1 [38] in the SUV-OE group (Fig. 6d, e), suggesting a suppressive effect of Suv39h1 on HOXA9 and MEIS1 signature genes. A suppression of E2F targets was also observed in the SUV-OE group (Fig. 6f), which was consistent with the phenotype of suppression of cell proliferation with SUV-OE (Fig. 4c–e). In addition, we found *Six1*, one of the MA9 targets [11, 39], was among the top of DEGs. Using qRT-PCR, we further validated the differential expression of several DEGs implicated in leukemogenesis, including *Six1*, *Hoxb13*, *Mlf1*, *Pax5* and *Dntt* (Fig. 6c). Genes involved in cell cycle regulation were also validated using qRT-PCR (Fig. 6g, h). These data indicated that Suv39h1 regulates leukemic transcriptional program in *MLL*-r leukemia.

### *Hoxb13* functions as a downstream effector of Suv39h1, and restoration of *Hoxb13* neutralizes the effect of SUV-OE

**Fig. 7:**
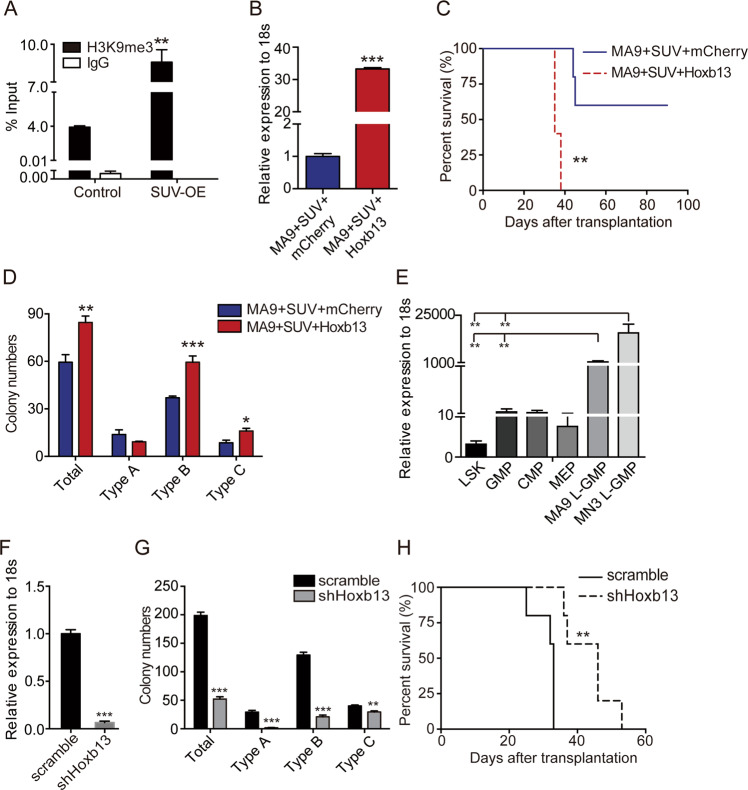
*Hoxb13* functioned as a downstream effector of SUV-OE and regulated MA9 AML progression. **a** H3K9me3 ChIP-qPCR analysis of *Hoxb13* promoter in c-Kit^+^ cells from control and SUV-OE AML mice. Data are presented as the means ± s.e.m., *n* = 3, **P < 0.01, Student’s *t* test. **b** Quantitative RT-PCR analysis of *Hoxb13* level in SUV-OE cells with Hoxb13-OE (MA9 + SUV + Hoxb13). Control cells were obtained in parallel with Hoxb13-OE cells (MA9 + SUV + mcherry). Data are presented as the means ± s.e.m., *n* = 3, ***P < 0.001, Student’s *t* test. **c** Kaplan–Meier survival curve of secondary recipients transplanted with leukemia cells; 1 × 10^3^ cells per group, *n* = 6, **P < 0.01, Mantel–Cox test. **d** Colony-forming assay of MA9 + SUV + Hoxb13 and control cells from secondary recipients; *n* = 4, *P < 0.05, **P < 0.01, ***P < 0.001, Student’s *t* test. **e** qRT-PCR analysis of *Hoxb13* level in normal murine HSPCs and leukemia stem cells from *MLL*-r leukemic mice. *n* = 3, *P < 0.05, **P < 0.01, Student’s *t* test. **f** *Hoxb13* expression levels in MA9 AML cells with mock shRNA or shHoxb13 (knockdown). *n* = 3, ***P < 0.001, Student’s *t* test. **g** Number of colonies of MA9 AML cells with *Hoxb13* knockdown (shHoxb13) or controls; *n* = 4, **P < 0.01, ***P < 0.001, Student’s *t* test. **h** Kaplan–Meier curves of secondary recipients transplanted with MA9-sh-scramble or with MA9-shHoxb13 cells, 33 days vs. 46 days, respectively; 1 × 10^4^ cells for each group (*n* = 10 per group), **P < 0.01, Log-rank (Mantel–Cox) test.

Given the transcriptional suppressive role of Suv39h1 by maintaining the transcriptional repressive modification mark H3K9me3, we focused on genes with significantly decreased expression in SUV-OE vs. control AML c-Kit^+^ cells. Among those leukemia-associated genes mentioned above, *Hoxb13* was of particular interesting because it is recurrently mutated in several types of cancers, including leukemia [40]. H3K9me3 ChIP-qPCR revealed that the H3K9me3 level at the promoter of *Hoxb13* was increased by twofold in SUV-OE groups (Fig. 7a), suggesting the decreased expression of *Hoxb13* might be a direct effect of the acquisition of H3K9me3 modification. By restoration of the *Hoxb13* expression in SUV-OE AML cells (Fig. 7b), we found an accelerated leukemia progression in SUV-OE AML mice, indicating that *Hoxb13* restoration abolished the effect of SUV-OE on prolonged survival of *MLL*-r AML mice (Fig. 7c). *Hoxb13* restoration also increased the colony formation capacity of SUV-OE cells (Fig. 7d). These data suggested a possible role of *Hoxb13* in leukemia progression.

### Dysregulation of *Hoxb13* affects MA9-induced AML progression

We next examined whether high expression of *Hoxb13* was an essential and common feature for MA9 AML progression. We found that *Hoxb13* was significantly overexpressed in LSCs from *MLL*-r AML mouse models as compared with that in HSPCs (Fig. 7e). Importantly, *HOXB13* exhibited increased expression in AML patient samples when compared with normal hematopoietic counterparts and the highest level in *MLL*-r patients when compared with other AML karyotypes (Supplementary Fig. 8a, b). Re-analysis of previously published ChIP-Seq data [11] revealed that the *Hoxb13* locus exhibited high levels of H3K79me2 and H3K4me3 and low levels of the repressive H3K27me3 in L-GMP than in LSK and GMP (Supplementary Fig. 8c). In addition, a lower level of H3K9me3 was also observed in c-Kit^+^ cells from MA9 and MN3 AML mice when compared with controls (Supplementary Fig. 8d, e). Thus, we reasoned that dysregulation of the epigenetic markers may contribute to the increased *Hoxb13* expression levels in MA9 LSCs. To further investigate the relationship of high *Hoxb13* expression and MA9 AML progression, we knocked down *Hoxb13* expression in MA9 leukemic cells (Fig. 7f). Indeed, knockdown of *Hoxb13* reduced their colony formation ability (Fig. 7g) and prolonged the survival of MA9 AML mice (Fig. 7h). Taken together, these data suggest that *Hoxb13* is epigenetically suppressed in normal HSPCs but activated in MA9 LSCs, and Suv39h1 functions in part by inactivation of *Hoxb13* to delay leukemic progression.

## Discussion

Although the interest in developing leukemia therapies by targeting H3K9 methyltransferases is increasing [41], the role of Suv39h1 and its associated H3K9me3 modification in *MLL*-r AML remains unclear. In the present study, we provide insight into the significance of Suv39h1 in regulating *MLL*-r leukemia and LSCs. We found that the expression of *SUV39H1* in AML blast cells and LSCs decreased as compared with their normal counterparts, and this correlated with overall outcomes of the disease. By restoration of the Suv39h1 expression, we demonstrated that this could decrease the number of functional LSCs and suppressed the development and progression of *MLL*-r AML. Correspondingly, we observed that suppression of Suv39h1 accelerated AML progression. Knockdown or inhibition of Suv39h1 led to the reduced H3K9me3 level, in the same trend as that Suv39h1 overexpression increased the H3K9me3 level. Our data thus strongly indicated Suv39h1 as a tumor suppressor for MA9 AML.

The expression of SUV39H1 was reported to decline with age in human and mouse HSCs, thus leading to a global H3K9me3 reduction and perturbed the heterochromatin function [16], and HSC aging increases the likelihood of progression to AML [17]. Our study provides evidences that reduced SUV39H1 expression may cause the disruption of the hematopoietic homeostasis and lead to malignant hematopoiesis. In leukemia cells of AML patients, it has been reported that H3K9me3 occupancy is reduced in the promoters containing cis-binding sites for ETS and cyclic adenosine monophosphate response elements (CREs) for CREB/CREM/ATF1, and decrease of H3K9me3 is correlated with event-free survival in AML patients with t(8;21), t(15;17), inv(16), or complex karyotype [28]. Our finding of decreased H3K9me3 level around TSS regions in *MLL*-r LSCs is in accordance with their observation of reduced H3K9me3 distribution in AML patients. Interestingly, they showed a correlation of decreased H3K9me3 with the CREB family in the study while we did not observe this correlation in our study, suggesting that there may be a different molecular mechanism underlying the differential distribution of H3K9me3 in distinct karyotypes of AMLs.

It has been reported that *Suv39h*-deficient mice spontaneously develop B-cell lymphomas at an increased frequency [42], and loss of Suv39h accelerates Ras- or Myc-driven tumorigenesis [43, 44]. Another study showed that increased Suv39h1 levels in *Suv39h1* transgenic mice impaired the clonogenic transduction potential of oncogenes Ras/E1A in primary MEF and displayed a resistance to 7,12-Dimethylbenz[α]anthracene/12-O-tetradecanoylphorbol-13-acetate (DMBA/TPA)-induced skin carcinogenesis [45]. These data, along with our findings demonstrate a tumor suppressive role of Suv39h1. However, SUV39H1 also participates in PML-RAR- [46] and EVI-1 [47]-mediated transcriptional repression and acts as an oncogenic cofactor of these two fusion genes to induce hematological malignancies. Thus, the role of SUV39H1 in cancer may be context-dependent, and its specific roles in other subtypes of leukemia require further investigation.

A subset of H3K9 methyltransferases including Suv39h1, G9a, GLP, and SETDB1 were reported to participate in one complex and cooperate functionally in gene silencing [48]. In consistent with this, our observation of the role of Suv39h1 in suppression of MLL-AF9 leukemia progression were in agreement with recent publication which reported a suppressive role of Setdb1 in *MLL*-fusion induced AML [24, 49]. Both studies revealed a decreased number of LSCs and increased apoptosis with H3K9 methyltransferase restoration. Interestingly, Suv39h1 and Setdb1 also shares some of the downstream targets, including reversing Hoxa9 and Meis1 signatures and suppressing MLL-AF9 target gene *Six1*. Nevertheless, pathway interaction analysis of DEG-enriched KEGG pathway revealed the reduction of metabolic process as one of the most prominent downstream effects of Suv39h1. Our recent study demonstrated that Six1 was a key transcription factor involved in leukemogenesis via regulation of LSC pools and the expression of glycolytic genes [36]. In addition, the expression of Six1 was decreased in SUV-OE cells, which suggests that a potential mechanism of Suv39h1 in MA9 AML progression may also be partially mediated by Six1.

Notably, Suv39h1 displayed a unique role in regulation cell cycle, which was not observed in Setdb1 OE AML cells. Retinoblastoma protein (Rb) is sequentially phosphorylated by CycD/Cdk4/6 and CycE/Cdk2 and full phosphorylated Rb activates the E2F transcription factor family, thus promotes the G1-S transition [50]. Previous study showed that Suv39h1 represses the expression of CycD1 to induce cell cycle arrest [51]. Here in this study, we found down-regulated expression of cell cycle activation genes (*Ccne1*, *Ccne2*, *Ccnd2* and E2F targets) as well as up-regulated expression of CDKi genes including *p18*, p21 family members *p21* and *p57* in SUV-OE cells. Thus, the decrease of S phase and the increase of G1 phase resulted by Suv39h1 overexpression were probably through coordinated regulation of G1/S transition related genes.

The recurrent mutation of G84E in the MEIS-interacting domain of HOXB13 [52] has been reported to be associated with leukemia and other cancers in a clinical correlation research [40]. Although a gain of function mechanism based on the lack of truncating mutations and the recurring nature of the G84E mutation has been suggested [52], the role of *Hoxb13* in AML progression has not been defined. Notably, the basal expression level of Hoxb13 is very low in normal hematopoietic cells, but is upregulated in MA9 LSCs. We demonstrated here that Hoxb13 itself was epigenetically dysregulated in MA9 cells, and it was highly activated in LSCs when compared with normal HSPCs. Moreover, knockdown of Hoxb13 prolonged the survival of MA9 AML mice. Interestingly, although HoxB13 shRNA knockdown did not affect the expression of other *HoxB* family gene, yet we did observe the down-regulated expression levels of some HoxA family members, such as *Hoxa9* and *Hoxa10* (Supplementary Fig. 9a, b). Both *HOXA9* and *HOXA10* were reported to be highly expressed and significantly correlated with leukemia progression and/or maintenance [53]. We reasoned that the lower expression levels of *HOXA9* and *HOXA10* probably were a reflection of slower progressing of leukemia rather than a HoxB13 shRNA non-specific knockdown effect on the HoxA family members. Our study thus showed that MLL-AF9 dependent expression of *Hoxb13* could be suppressed by SUV39H1. Furthermore, restoration of Hoxb13 in SUV-OE AML cells accelerated the leukemia progression and increased the number of LSCs, suggesting *Hoxb13* is a downstream effector of Suv39h1 in MA9 leukemia cells.

Herein, we demonstrate that *Suv39h1* is significantly down-regulated in AMLs and could function as a tumor suppressor in *MLL*-rearrangement induced leukemia by regulating *Hoxb13* and *Six1*, as well as Hoxa/Meis1 downstream signature genes. The underlying molecular mechanism mediated by the Suv39h1 in tumor suppression may provide a potential novel therapeutic targeting strategy for *MLL*-rearranged leukemia.

## Materials and methods

### Human samples and ethics

Primary human AML blasts and cord blood were obtained from an experimental pathology cell bank of the State Key Laboratory of Experimental Hematology (SKLEH) from Institute of Hematology and Blood Diseases Hospital (IHBD). Sample acquisition was approved by the Institutional Review Boards at the IHBD. CD34^+^ cells were enriched using a CD34 MicroBead Kit (Miltenyi, Bergisch Gladbach, Germany). Detailed information of patient samples was described previously [54].

### Mice

Wild-type (WT) C57BL/6J mice were acquired from the animal facility of SKLEH. 8-week-old female mice were used for the experiments. Same batches of mice were randomly assigned to control and experiment groups. All animal procedures were performed in accordance with the guidelines of the Institutional Animal Care and Use Committee of the IHBD, Chinese Academy of Medical Sciences.

### Establishment of in vivo mouse models

MLL-AF9 (MA9) and MLL-NRIP3 (MN3) AML mouse models were established as described previously [55, 56]. MN3 was a relatively new *MLL* translocation reported by Balgobind et al. [57], and the mouse model was established in our hospital [56, 58]. Serial transplantation was performed to determine the self-renewal potential of the established MA9 leukemia cells, and P3 cells were used for all subsequent experiments for consistency. Full scan images of Western blots are supplied as Supplementary Fig. 10. The antibodies used for Western blots are listed in Supplementary Table 1.

### Limit dilution assay

Series doses of leukemic cells were collected from the BM of suv39h1 overexpression (SUV-OE) or experimental eGFP^+^ control mice and subjected to a limiting dilution series. The frequencies of LSCs were calculated according to Poisson statistics using ELDA software [59].

### RNA-seq and chromatin immunoprecipitation (ChIP)-seq

Enriched c-Kit^+^ cells from moribund SUV-OE or control mice were used. All procedures were performed as described previously [58]. The data have been submitted to the NCBI Gene Expression Omnibus under accession number GSE115549 for RNA-seq and GSE132175 for ChIP-seq. The primers used in this study have been supplied in the Supplementary Table 2.

### Statistical analysis

All experiments were repeated two to three time with the indicated numbers of mice. Sample size for each experiment was determined according to experience or the previously published papers. The investigator was not blinded during experiment or assessing the outcome. Distribution was tested using the modified Shapiro–Wilks method. When parameters followed Gaussian distribution, Student’s *t* test was used for two groups’ analyses and one-way ANOVAS was for comparing more than two groups to evaluate the statistical significance. We have statistically compared the similar variance between the groups as well. Analyses were carried out using the Prism version 7.01 software (La Jolla, CA, USA).

## Supplementary information

Supplemental material
