# Supplementary material for: SUV39H1 regulates the progression of MLL-AF9-induced acute myeloid leukemia
Source: Oncogene. 2020 Oct 9;39(50):7239–52. doi: 10.1038/s41388-020-01495-6 (PMC7728597; doi:10.1038/s41388-020-01495-6)
Supplement: Supplementary file 1 — Supplemental material [file 41388_2020_1495_MOESM1_ESM.pdf]

Supplementary file for

Chu *et al.*, "SUV39H1 regulates the progression of *MLL-AF9*-induced acute myeloid leukemia"

Table of contents:

1. Supplementary Materials and Methods

2. Supplementary Figures 1-10

Supplementary Figure 1. Suv39h1 expression analyses and its correlation with prognosis

Supplementary Figure 2. H3K9me3 ChIP-seq analyses of c-Kit$^+$ cells from AML or WT control mice

Supplementary Figure 3. Establishment of Suv39h1-overexpressing (SUV-OE) MA9 AML mouse model and its phenotype analyses

Supplementary Figure 4. The effects of SUV39H1 overexpression and knockdown on cell proliferation and apoptosis in AML human cell lines bearing MLL-fusion

Supplementary Figure 5. Analyses of chaetocin treatment in AML mouse model

Supplementary Figure 6. Suppression of Suv39h1 accelerated AML progression.

Supplementary Figure 7. Pathway analyses of differentially expressed genes of control and SUV-OE c-Kit$^+$ cells

Supplementary Figure 8. Epigenetic regulation of *Hoxb13* in MA9-induced AML and aberrant expression of *HOXB13* in human patients

Supplementary Figure 9. The expression levels of HoxB or HoxA family genes with Hoxb13 shRNA knockdown

Supplementary Figure 10. Full scan images of Western blots for SUV-OE and shSUV cells

3. Supplementary Table 1-2

Supplementary Table 1. Antibodies or related reagents used in the study

Supplementary Table 2. qRT-PCR and ChIP-qPCR primers used in the study

4. Supplementary References 1-4

1. Supplementary Materials and Methods

Immuno-blotting analysis

Leukemic bone marrow cells or c-Kit$^+$ cells were lysed with 1× SDS sample buffer for 30 min at room temperature, boiled at 100°C for 10 min, and stored at -20°C for use. Samples of equal amount of protein were separated by SDS-PAGE, transferred on a PVDF membrane (Millipore), blocked with fat-free milk, and then incubated with primary antibodies. After washing, blots were incubated with HRP-conjugated secondary antibody for 1h. Signals were detected with the Immobilon Western Chemiluminescent HRP substrate (Millipore). Densitometry was performed and calculated with ImageJ (1.49v, NIH). The antibodies used for immune-blotting are listed in Supplementary Table 1.

Cell line culture and CCK8 analysis

Human cell lines HEK293T, THP-1, Molm13 and Nomo-1 cells were obtained from cell bank of SKLEH (purchased from ATCC and maintained by cell bank). The cells were tested for mycoplasma contamination monthly using a mycoplasma detection kit (InvivoGen). Cell lines were validated for authentication using the short tandem repeat method (Beijing Microread Corporation Limited). THP-1, Molm13 and Nomo-1 were cultured at 4-6×10^5 cells/ml in RPMI-1640 GlutaMAX containing 10% FBS, 25 mM HEPES, 100 U/ml penicillin, and 100 μg/ml streptomycin. Cells were transduced with lentiviruses expressing SUV39H1-eGFP, and 48h after infection, GFP$^+$ cells were sorted with flow cytometry and collected. Cells were then seeded in 96-well plates at a density of 5000 cells/well in 100 µl volume. Ten µl of Cell Counting Kit-8 (Dojindo) were added to each well and allowed to react with cells for 4h. The absorbance was read at 450 nm by Synergy H4 Microplate Reader (Biotek).

Colony forming capacity (CFC) assays for mouse cells

Leukemic cells (eGFP$^+$ for SUV39H1 overexpression) were sorted, and cultured in methylcellulose-based medium (3231, STEMCELL Technologies) supplied with 10 ng/mL mIL-6, 10 ng/mL mIL-3, 50 ng/mL mSCF and 10 ng/mL mGM-CSF (Peprotech) and 100U/mL Penicillin-Streptomycin. The cells were plated in 24-well plates in a 0.5 ml volume at 400 cells per well. CFCs were scored under an inverted microscope after 7-10 days' culture. In the replating CFC assays, 500 cells were

plated in each well in the first-round CFC. For replating assay, colony cells were collected and replated in methylcellulose every 6 days with 500 cells as input for each passage (number of colonies at the 3rd plating were calculated).

Plasmids construction

Suv39h1 was amplified from mouse BM cDNA with primers (forward primer: atgggggagccggcgactctaggttgcre, reverse primer: ctgaagcggtattttcggcaagcc), and inserted into MSCV-IRES-EGFP plasmid via *Eco*RI site. *Hoxb13* was synthesized and inserted into pCDH1-MSCV-EF1-mCherry plasmid via *Eco*RI and *Bam*HI site by Hanbio company. Primers and shRNA targeting *Suv39h1* (mature sha RNA: TGTAATCAAAGGTGAGCTC, mature shb RNA: ACAGCACACACTGCAACCT) and *Hoxb13* (mature RNA: ATCATGACAACTAGTACTG) was synthesized by BGI tech company, annealed and then inserted into SF-LV-shRNA-BFP plasmid.

Apoptosis assay

The apoptosis assay was performed as previously described [1]. Briefly, leukemic mouse BM or spleen cells were labeled with surface antibodies. Cells were washed with 1 × PBS, resuspended in 1 × Binding Buffer (BD Bioscience, Carlsbad, CA, USA) and stained with Annexin V and 7-AAD (BD Bioscience, Carlsbad, CA, USA) according to the manufacture manual. Cells were analyzed using a CANTO II flow cytometer (BD Bioscience, USA).

Cell cycle analysis

For cell cycle analyses, leukemic mouse BM or spleen cells were labeled with surface antibodies, fixed and permeabilized with BD IntraSure Kit (BD Bioscience, San Jose, CA, USA). Cells were intracellularly stained with antibodies against Ki67 (BD Bioscience, Carlsbad, CA, USA) and Hoechst 33342 (Sigma-Aldrich), and measured using the CANTO II flow cytometer (BD Bioscience, USA). BrdU incorporation assay was used to analyze cell cycle. Cells were incubated with BrdU (1 mM) for 1-2 hours before detecting. Cells were fixed, permeabilized and stained with anti-BrdU antibody and 7-AAD by using BD Pharmingen™ BrdU Flow Kits (BD Bioscience, San Jose, CA, USA). Cells were measured by the CANTO II flow cytometer (BD Bioscience, USA).

Lentivirus preparations and bone marrow transplantation

All procedures were performed as described previously[1]. $EGFP^+$, $mCherry^+$ or BFP^+ cells were sorted and transplanted into sub-lethally (4.5 Gy) irradiated mouse recipients.

Droplet digital PCR (ddPCR)

The QX200 droplet digital PCR System (Bio-Rad) was used to detect the target genes in a duplex reaction. Evagreen PCR reaction mixture was assembled from a 2×ddPCR Mastermix (Bio-Rad), 20×primer (final concentrations of 100 nM, respectively) and template (Roughly 20 ng of cDNA) in a final volume of 20 μL. We partitioned each reaction mixture into approximately 20 000 droplets with a droplet generator (Bio-Rad) and then cycled with the following conditions: 95℃×10 min (1 cycle), 94℃×30 s and 60℃×1 min (40 cycles), 98℃×10 min (1 cycle). Primers used in ddPCR were validated by qRT-PCR. QuantaSoft analysis software (Bio-Rad) was used to calculate the absolute quantification of the target genes.

BrdU incorporation assay

Mice were intraperitoneal injected with 1 mg of BrdU 2h before they were sacrificed. BM cells were harvested and stained with fluorescent-conjugated antibodies. Labeled BM cells were fixed, permeabilized, and treated with DNase to expose the incorporated BrdU using a BrdU Flow Kit (BD Biosciences). Cells were then stained with an APC-labeled anti-BrdU and analyzed using a CANTO II flow cytometer.

RNA-seq and ChIP-seq analysis

RNA-seq libraries were generated using the Illumina TruSeq RNA sample preparation kit v2 following manufacturer's protocol. The libraries were sequenced on an Illumina Hiseq 2000 platform using a 150-bp paired-end sequencing strategy. Read alignment against mouse genome reference (mm10) was performed using TopHat2[2]. The FPKM (fragments per kilobase of transcript per million mapped reads) value of transcripts were calculated by the Cufflink algorithm[3] representing the gene expression level. Differentially enriched genes were enriched with DESeq2. ChIPed DNA was subjected to library construction according to Illumina's TruSeq® ChIP

Sample Preparation according to the protocol. 50-cycle single-read sequencing was performed using an Illumina HiSeq 2500.

2. Supplementary Figures 1-10

Supplementary Figure 1

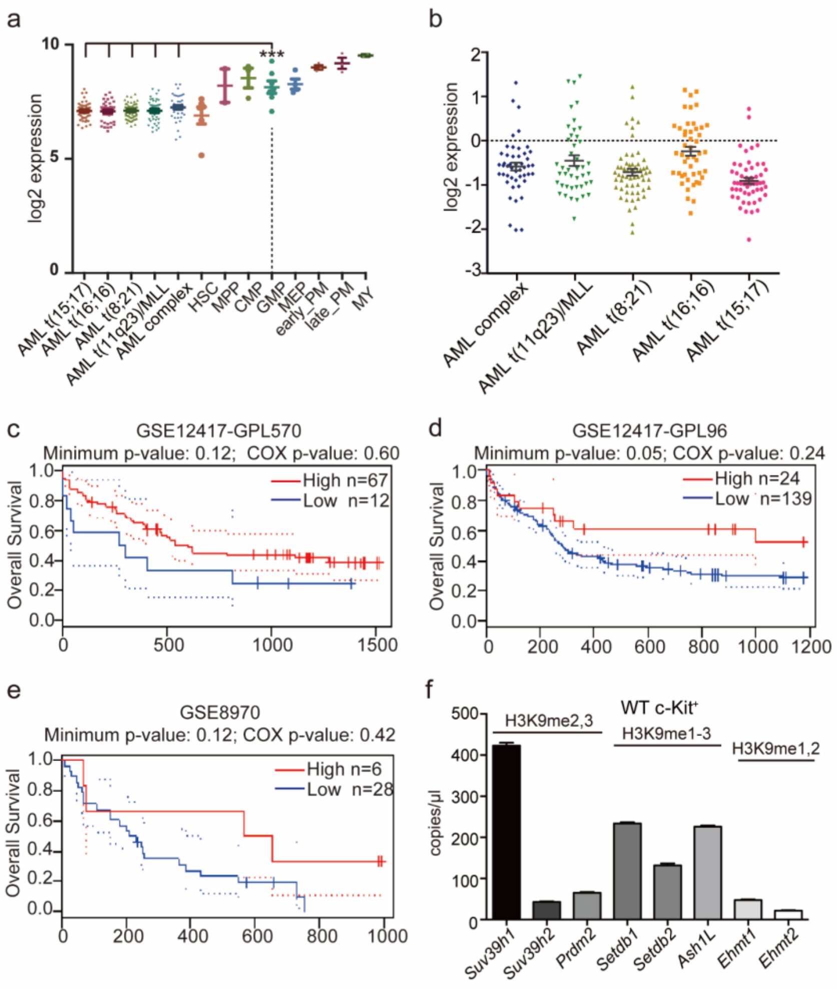

Supplementary Figure 1. Suv39h1 expression analyses and its correlation with prognosis

(**a**, **b**) Expression patterns of *SUV39H1* in normal and malignant human hematopoietic cells (GSE42519 and GSE13159 for **a**, GSE6891 for **b**, collected in Bloodspot). ***$P < 0.001$, Student's *t* test.

(**c-e**) Comparison of overall survival (OS) between AML patients with higher or lower levels of *SUV39H1* expression (grouped according to the median expression using PrognoScan, Mantel-Cox test).

(**f**) Droplet digital PCR analysis of expression levels of H3K9 methyltransferases in WT BM c-Kit^{+} cells.

Supplementary Figure 2

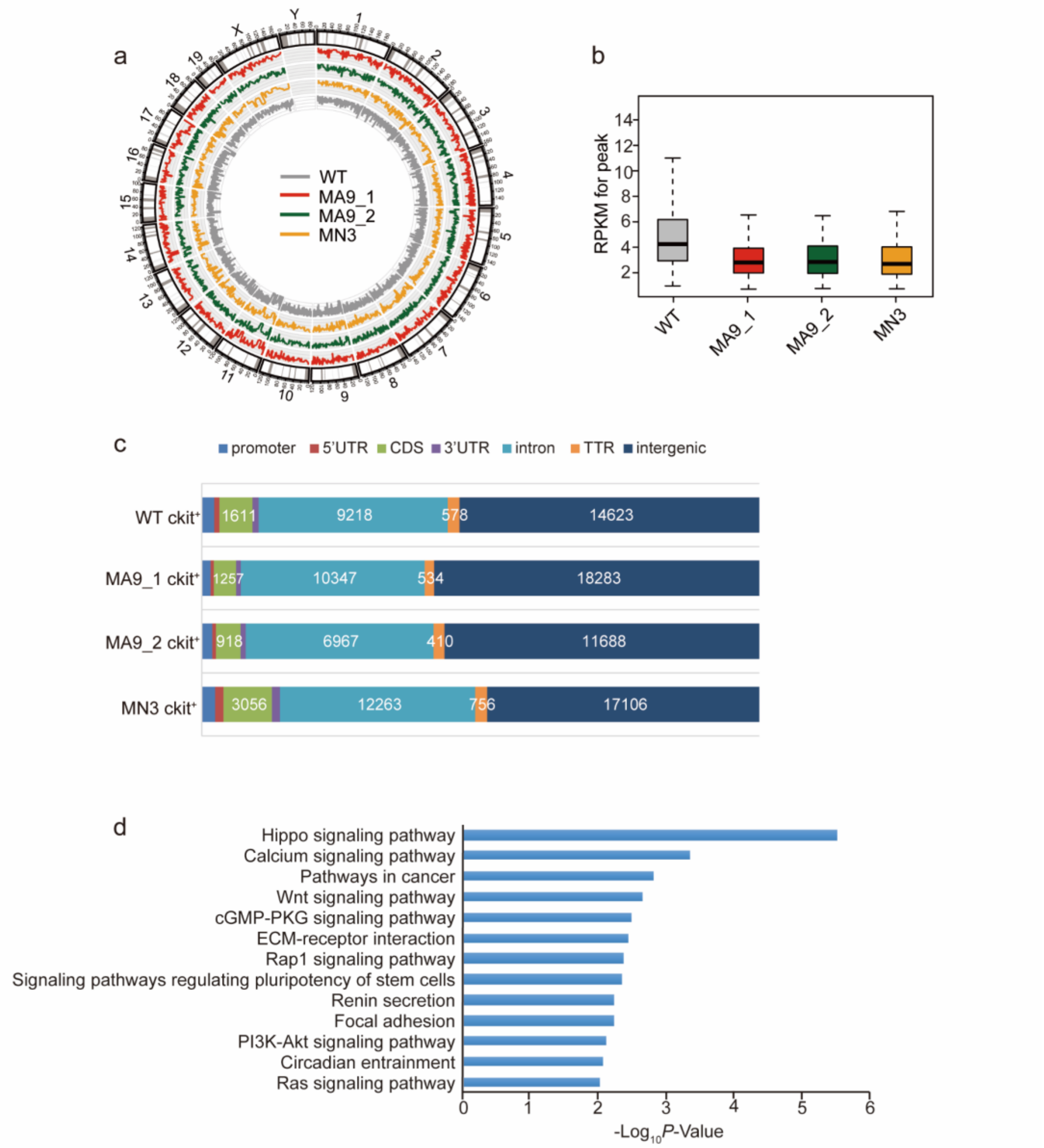

Supplementary Figure 2. H3K9me3 ChIP-seq analyses of c-Kit+ cells from AML or WT control mice

(**a**) Circos maps showing genomic distribution for the ChIP-seq peaks of H3K9me3 in c-Kit+ cells isolated from wild-type (WT) or MLL-rearranged gene (MLL-r)-induced AML mice. The track height denotes the significance of the peak, that is, the -log10 (P-value of peak); MA9, MLL-AF9; MN3, MLL-NRIP3.

(**b**) Box plots showing RPKM (reads per kilobase per million mapped reads) values for ChIP-seq peaks of H3K9me3 in c-Kit+ cells isolated from WT or MLL-r leukemic mice.

(**c**) Distribution of H3K9me3 enriched regions with genomic features in c-Kit+ cells isolated from leukemia or WT control mice.

(**d**) KEGG pathway analysis of H3K9me3 differentially enriched genes.

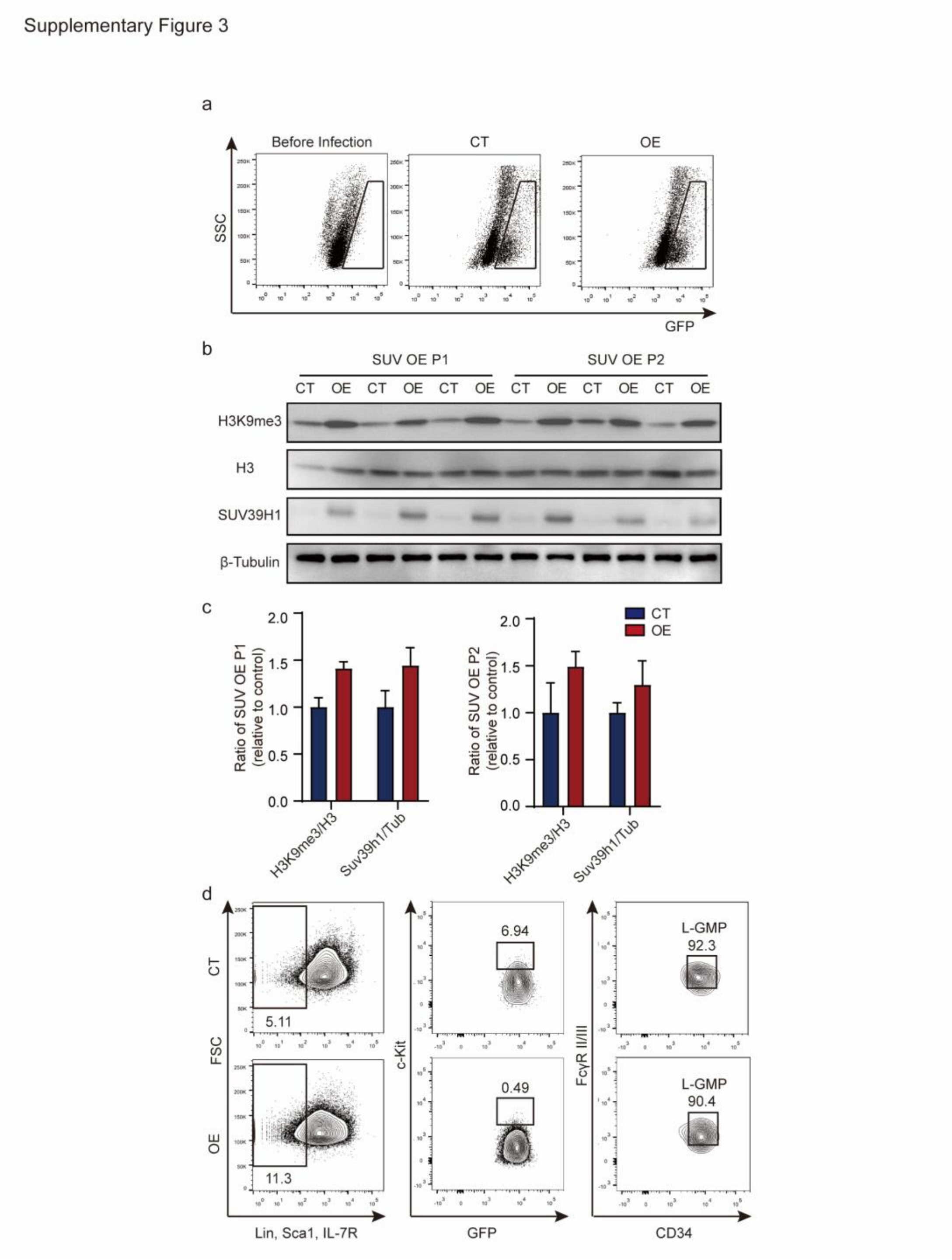

Supplementary Figure 3. Establishment of Suv39h1-overexpressing (SUV-OE) MA9 AML mouse model and its phenotype analyses

(**a**) Representative flow cytometric analysis of eGFP^{+} BM cells isolated from the control (CT) and SUV-OE mice.

(**b**) Immunoblot analyses of H3K9me3 and Suv39h1 levels in whole BM cells from control and SUV-OE P1 or P2 recipients.

(**c**) The densitometry of relative Suv39h1 and H3K9me3 levels in SUV OE groups (P1 and P2) was normalized to β-tubulin and H3, respectively. Densitometry was calculated with ImageJ, n =3 for each group.

(**d**) Representative flow cytometric analysis of L-GMP in BM cells isolated from control (CT) and SUV-OE mice.

Supplementary Figure 4

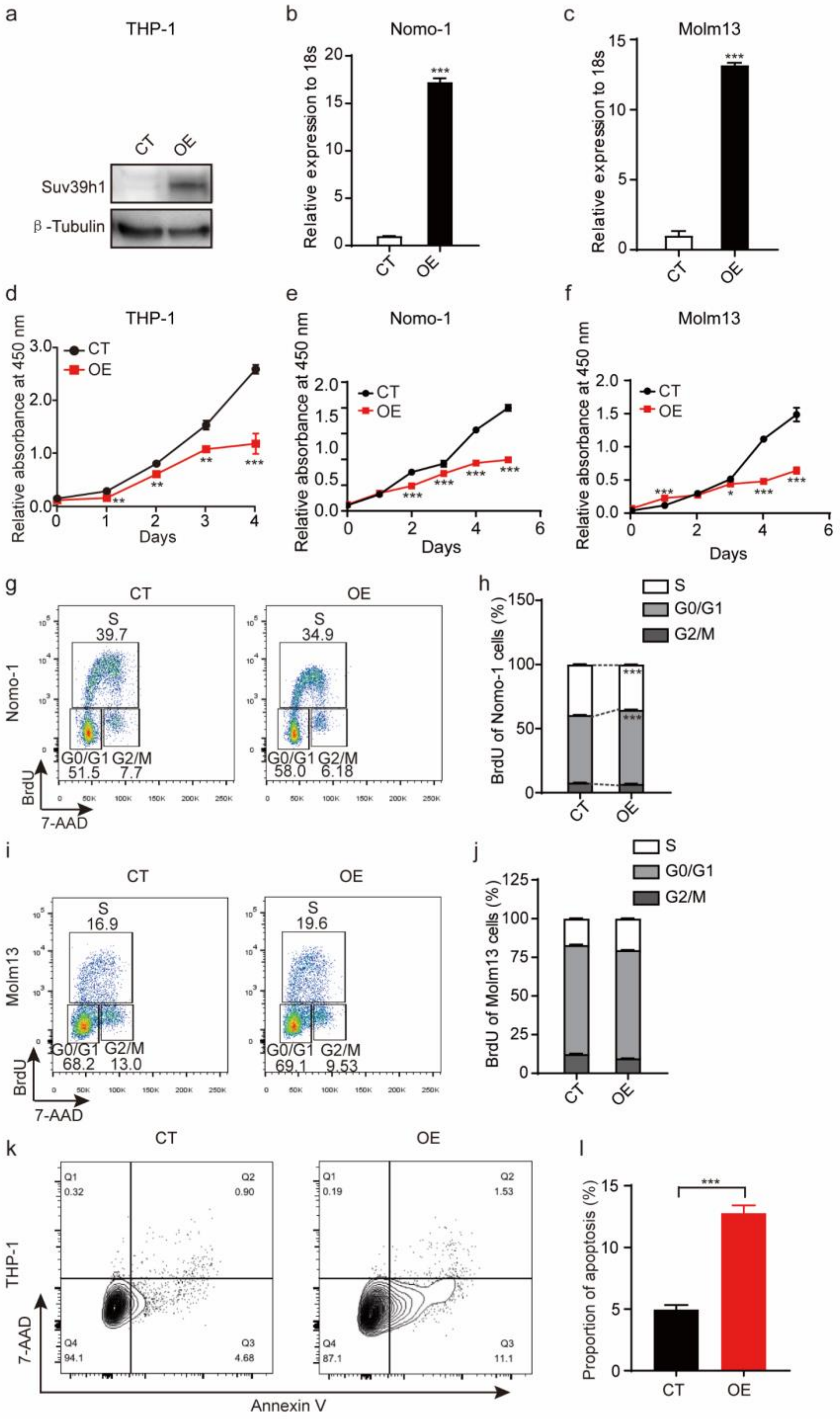

Supplementary Figure 4 continued

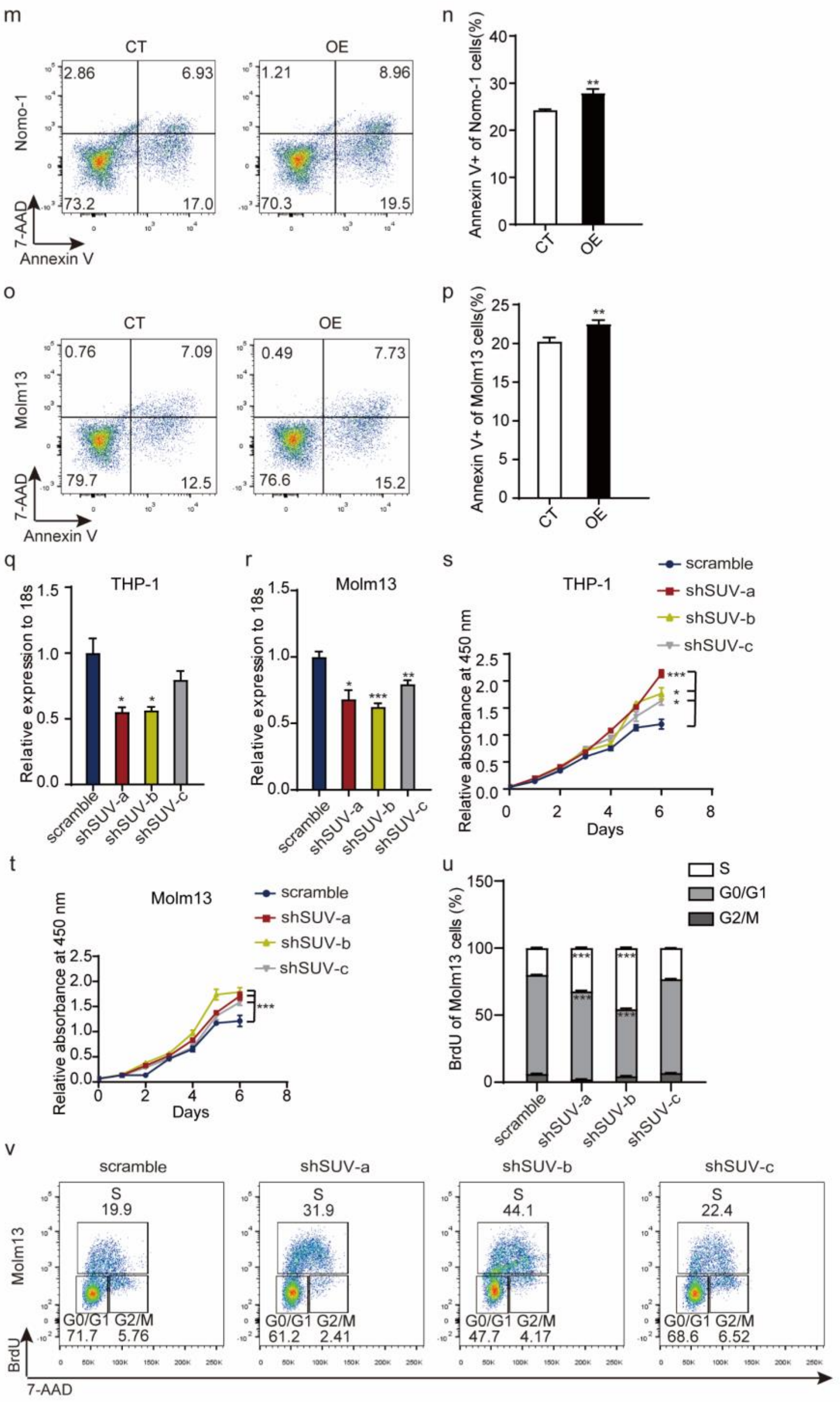

Supplementary Figure 4. The effects of SUV39H1 overexpression and knocking down on cell proliferation and apoptosis in AML human cell lines bearing MLL-fusion

(**a**) Immunoblot of SUV39H1 in AML stable cell line THP-1 transduced with control (CT) or *SUV39H1*-expression lentivirus.

(**b, c, q and r**) Quantitative RT-PCR analysis of *Suv39h1* level in cell lines (b for SUV-OE Nomo-1cells, c for SUV-OE Molm13 cells, q for shSUV THP-1 cells and r

for shSUV Molm13 cells). Data of b and c are presented as the means ± s.e.m., n = 3, ****P* < 0.001, Student's *t* test. Data of q and r are presented as the means ± s.e.m., n = 3, **P* < 0.05, ***P* < 0.01, ****P* < 0.001, by one-way ANOVA.

(**d, e, f, s and t**) Proliferation curves of control and SUV-OE or shSUV cell lines (d for SUV-OE THP-1 cells, e for SUV-OE Nomo-1 cells, f for SUV-OE Molm13 cells, s for shSUV THP-1 cells and t for shSUV Molm13 cells) were measured by CCK8, Data of d, e and f are presented as the means ± s.e.m., n = 3 for d, n=4 for e and f, **P* < 0.05, ***P* < 0.01, ****P* < 0.001, Student's *t* test. Data of s and t are presented as the means ± s.e.m., n = 4, **P* < 0.05, ****P* < 0.001, by one-way ANOVA.

(**g, i and v**) Representative flow cytometric analysis of cell cycle of cell lines (g for SUV-OE Nomo-1 cells, i for SUV-OE Molm13 cells and v for shSUV Molm13 cells).

(**h, j and u**) Quantification of G0/G1, S and G2/M phases of cell lines (h for SUV-OE Nomo-1 cells, j for SUV-OE Molm13 cells and u for shSUV Molm13 cells). n=5 for SUV-OE Nomo-1 cells and SUV-OE Molm13 cells. ****P* < 0.001, Student's *t* test. n=3 for shSUV Molm13 cells, ****P* < 0.001, by one-way ANOVA.

(**k, m and o**) Representative flow cytometric analysis of apoptosis of cell lines (k for SUV-OE THP-1 cells, m for SUV-OE Nomo-1 cells and o for SUV-OE Molm13 cells).

(**l, n and p**) Statistical analysis of apoptotic ratio of cell lines (l for SUV-OE THP-1 cells, n for SUV-OE Nomo-1 cells and p for SUV-OE Molm13 cells) in comparison with controls. n = 3, ***P* < 0.01, ****P* < 0.001, by Student's *t* test.

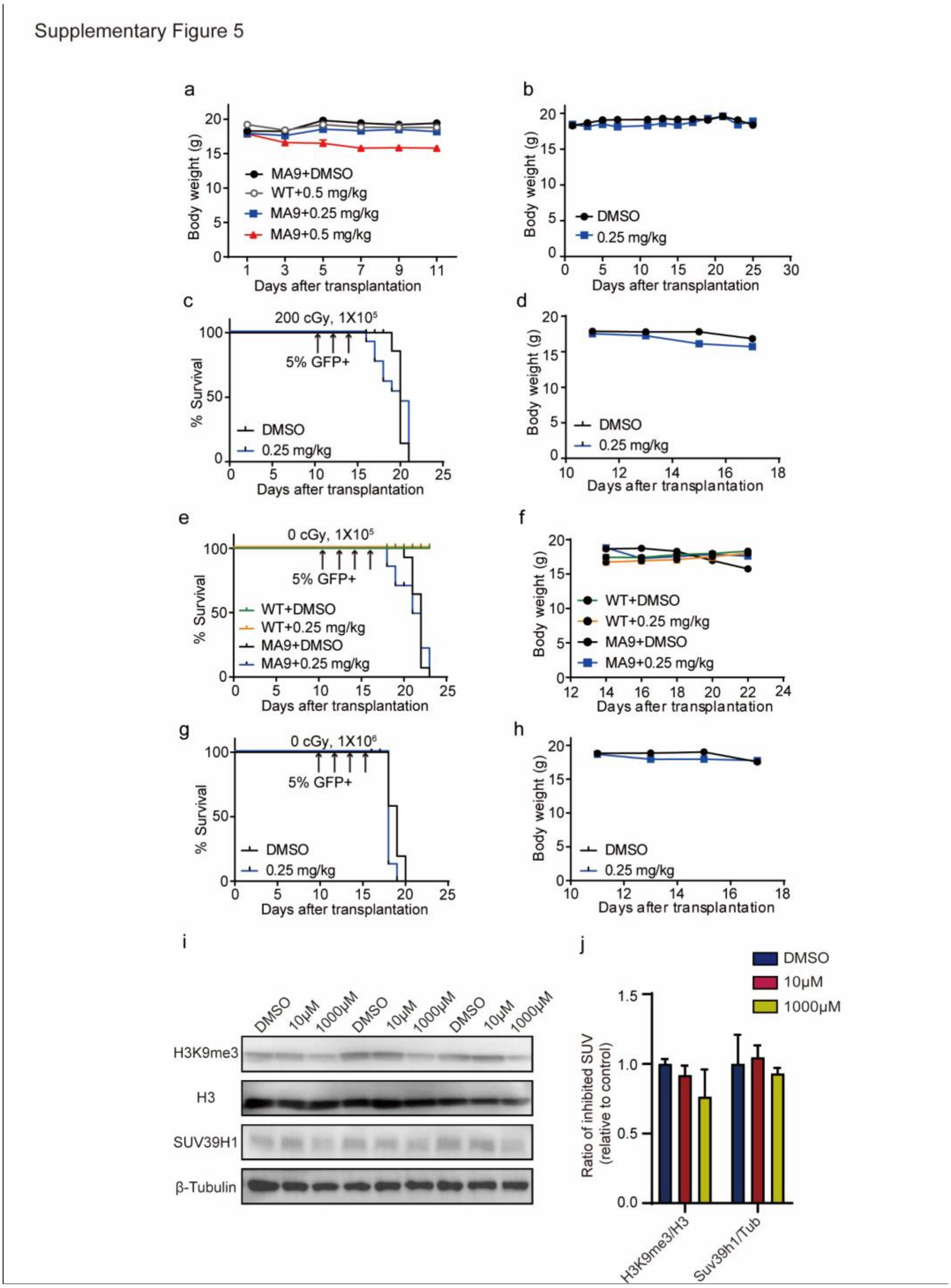

Supplementary Figure 5. Analyses of chaetocin treatment in AML mouse model

(**a**) Body weight monitoring for mice in Figure 5B.

(**b**) Body weight monitoring for mice in Figure 5C.

(**c**) Kaplan–Meier survival curve. Mice were sub-lethally irradiated and transplanted with 1×10^5 MA9 P2 AML cells. Treatment started when the percentage of GFP^+ cells in peripheral blood reached to about 5%. Intraperitoneal (ip) injections were done every two days. n =7 for DMSO group, n=8 for chaetocin group. Mantel-Cox test, statistically non-significant.

(**d**) Body weight monitoring for mice in panel c.

(**e**) Kaplan–Meier survival curve of non-irradiated mice transplanted with 1×10^5 MA9 P2 AML cells. Treated as in panel c. n =10 for WT group, n =14 for DMSO group, and n=15 for chaetocin group. Mantel-Cox test, statistically non-significant.
(**f**) Body weight monitoring for mice in panel e.
(**g**) Kaplan–Meier survival curve of non-irradiated mice transplanted with 1×10^6 MA9 P2 AML cells. Treated as in panel c. n =7 for DMSO group, n =8 for chaetocin group. Mantel-Cox test, statistically non-significantstatistically non-significant.
(**h**) Body weight monitoring for mice in panel g.
(**i**) Immunoblot analysis of Suv39h1 and H3K9me3 levels in whole BM cells from control and chaetocin treated (10 μM and 1000 μM, 24h).
(**j**) The densitometry of relative Suv39h1 and H3K9me3 levels in chaetocin treated groups (10 μM and 1000 μM) was normalized to β-tubulin and H3, respectively. Densitometry was calculated with ImageJ, n =3 for each group.

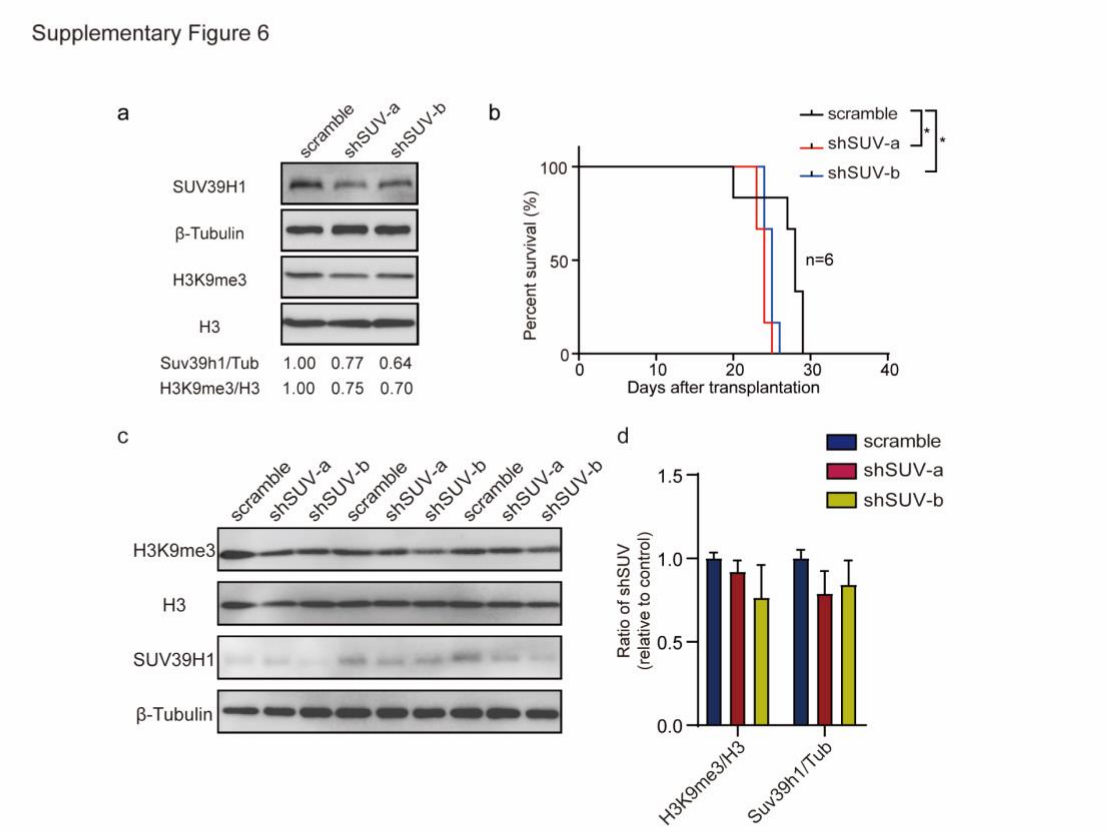

Supplementary Figure 6. Suppression of *Suv39h1* accelerated AML progression.
(**a**) Immunoblot analyses of Suv39h1 and H3K9me3 levels in whole BM cells from re-established scramble, shSUV-a or shSUV-b recipients. Densitometry calculated with ImageJ.
(**b**) Kaplan–Meier survival curves of secondary (transplanted with re-established P0 cells, 2×10^4 cells per group, shSuv-a and shSuv-b) recipients (n=6). Mantel-Cox test, *$P < 0.05$.
(**c**) Immunoblot analysis of Suv39h1 and H3K9me3 levels in whole BM cells from control, shSUV-a or shSUV-b primary recipients.
(**d**) The densitometry of relative Suv39h1 and H3K9me3 levels in in shSUV groups (shSUV-a and shSUV-b) was normalized to β-tubulin and H3, respectively. Densitometry was calculated with ImageJ, n =3 for each group.

Supplementary Figure 7

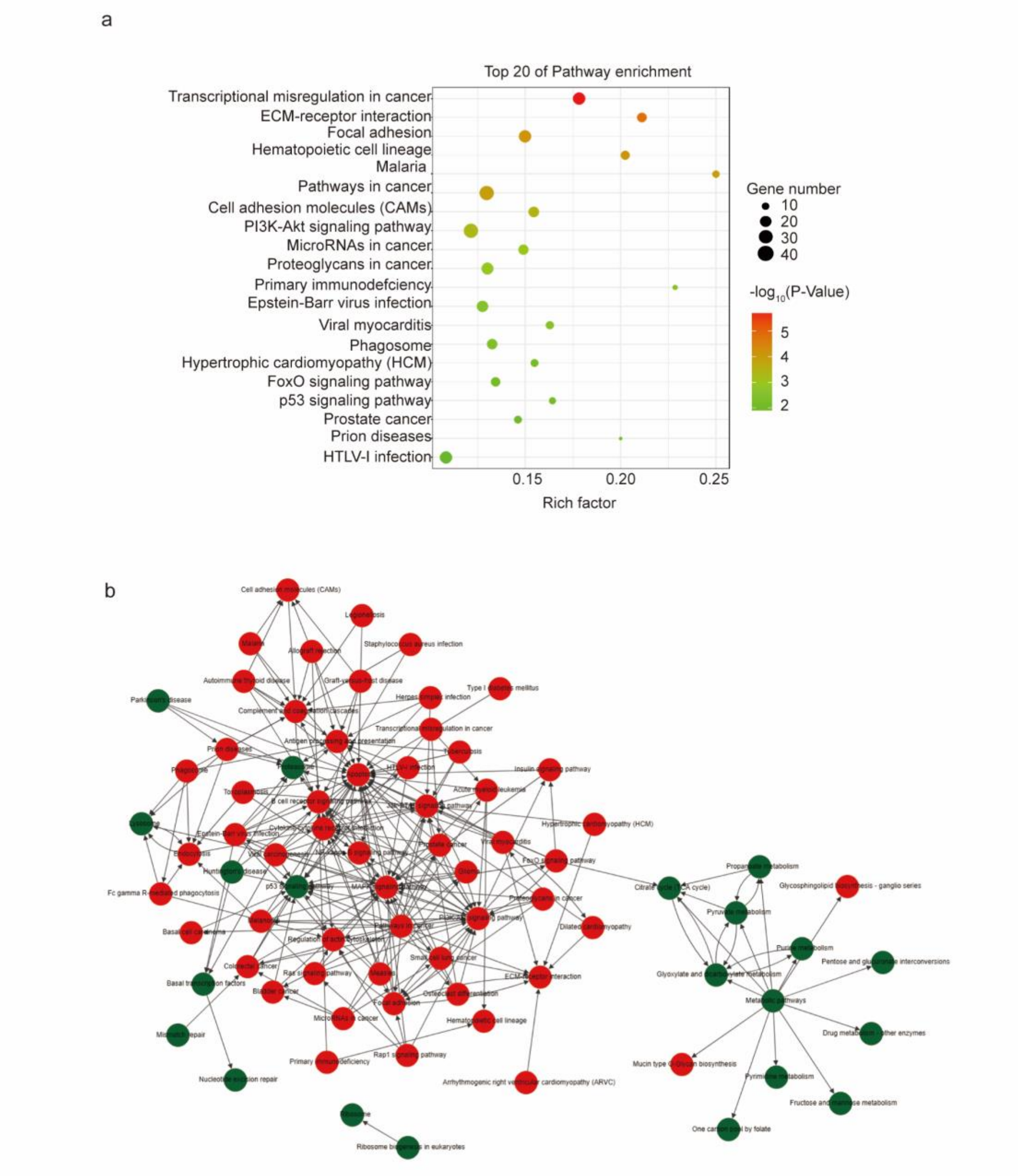

Supplementary Figure 7. Pathway analyses of differentially expressed genes of control and SUV-OE c-Kit$^+$ cells

(**a**) Top 20 of KEGG pathway enrichment of DEGs. Rich factor represents the ratio of the enriched DEGs number in this pathway to the number of genes annotated in this pathway.

(**b**) DEG enriched KEGG pathway interaction analysis by Cytoscape. Red color represents the significantly enriched pathway in upregulated genes in SUV-OE groups, whereas green color represents the significantly enriched pathways in down-regulated genes in SUV-OE groups. Arrow source indicates upstream and arrow target indicates downstream.

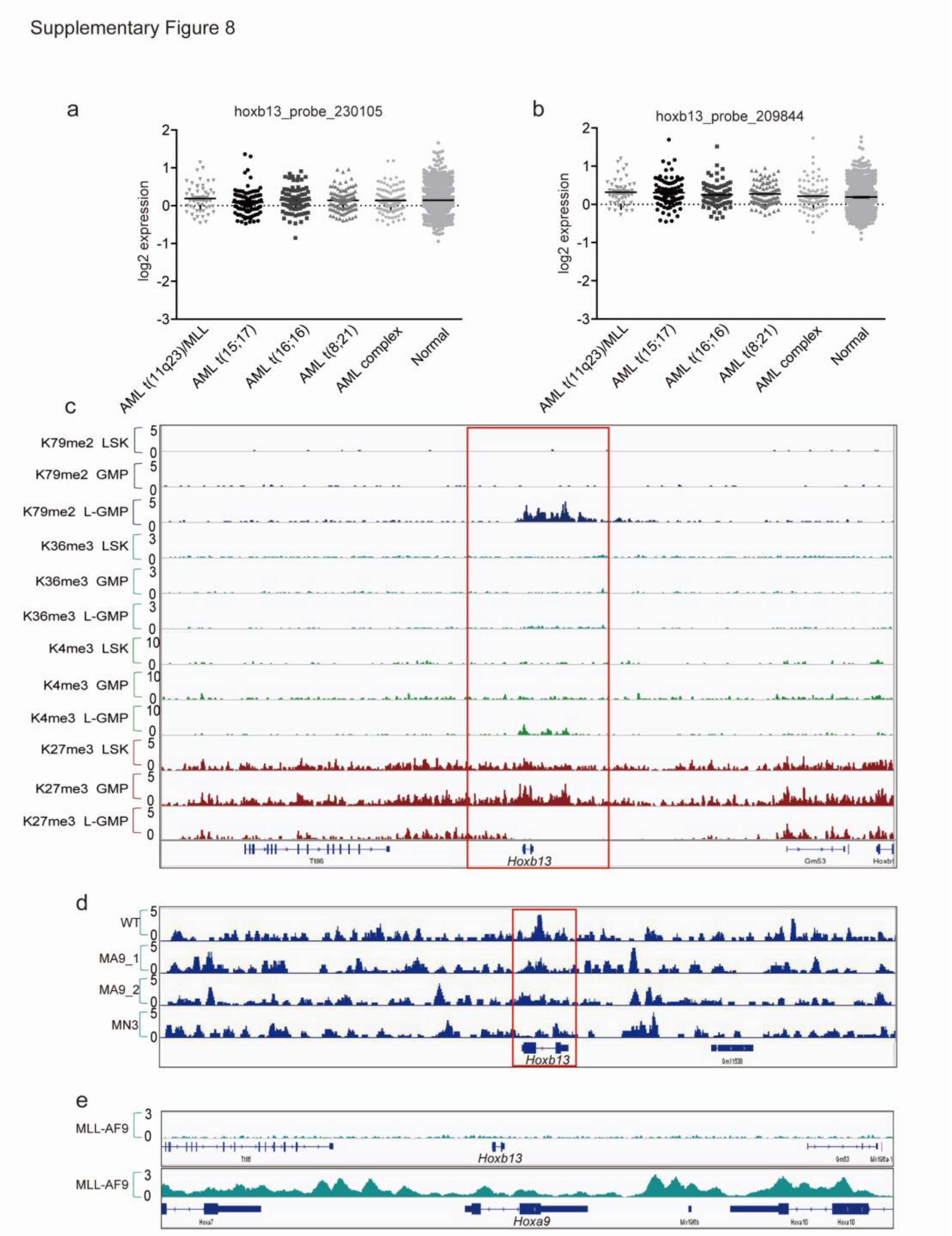

Supplementary Figure 8. Epigenetic regulation of *Hoxb13* in MA9-induced AML and aberrant expression of HOXB13 in human patients

(**a, b**) Expression patterns of *HOXB13* in AML patients normalized to the nearest normal hematopoietic counterpart (a and b with different probes, collected in Bloodspot). ***$P < 0.001$, Student's t test.

(**c**) Re-analysis of published ChIP-seq data [4] for H3K79me2, H3K36me3, H3K4me3 and H3K27me3 modifications of *Hoxb13* in sorted LSK, GMP and L-GMP populations in MLL-AF9 transformed murine cells.

(**d**) H3K9me3 distribution across the Hoxb13 loci in enriched c-Kit^{+} cells from WT, MA9 and MN3 BM cells.

(**e**) Re-analysis of public MLL-AF9 ChIP-seq data [4] in *MLL-AF9* transformed cells.

Supplementary Figure 9

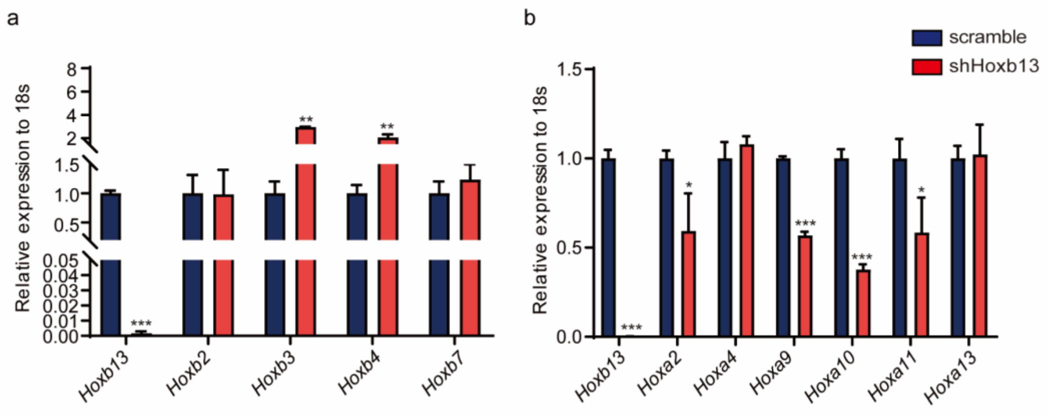

Supplementary Figure 9. The expression levels of HoxB or HoxA family genes with *Hoxb13* shRNA knockdown

(**a**) Expression levels of *HoxB* family in control (scramble) and shHoxb13 groups. Data are presented as the means ± s.e.m., n = 3, $^{**}P < 0.01$, $^{***}P < 0.001$, Student's *t* test.

(**b**) Expression levels of *HoxA* family in control (scramble) and shHoxb13 groups. Data are presented as the means ± s.e.m., n = 3, $^{*}P < 0.05$, $^{***}P < 0.001$, Student's *t* test.

Supplementary Figure 10

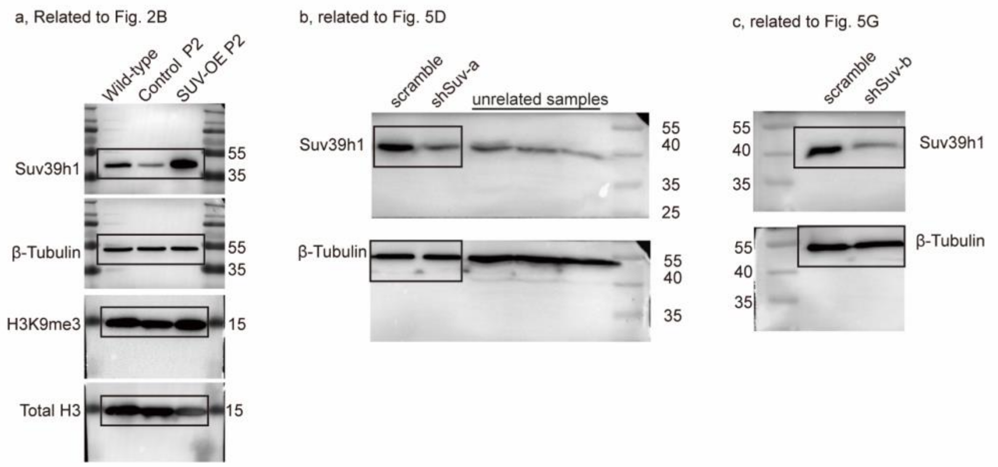

Supplementary Figure 10. Full scan images of Western blots for SUV-OE and shSUV cells

(**a**) Immunoblot analysis of Suv39h1 and H3K9me3 levels in whole BM cells from control or SUV-OE tertiary recipients.

(**b, c**) Immunoblot analyses of Suv39h1 level in whole BM cells from control or shSUV primary recipients (**b** for shSuv-a and **c** for shSuv-b).

3. Supplemental Table 1-2

Supplementary Table 1. Antibodies or related reagents used in the study

Antibodies or reagents	Vendor	Cat. No.	Dilution	Application
anti-CD3	eBioscience	145-2C11	0.5 µg/10^6 cells	FACS
anti-CD4	eBioscience	GK1.5 or RM4-5	0.25 µg/10^6 cells	FACS
anti-CD8a	eBioscience	53-6.7	0.5 µg/10^6 cells	FACS
anti-B220	eBioscience	RA3-6B2	1 µg/10^6 cells	FACS
anti-Mac-1	eBioscience	M1/70	0.06 µg/10^6 cells	FACS
anti-Gr-1	eBioscience	RB6-8C5	0.125 µg/10^6 cells	FACS
anti-Ter-119	eBioscience	TER-119	0.25 µg/10^6 cells	FACS
anti-Sca-1	eBioscience	D7	0.5 µg/10^6 cells	FACS
anti-c-Kit	eBioscience	2B8	0.5 µg/10^6 cells	FACS
anti-CD34	eBioscience	RAM34	1 µg/10^6 cells	FACS
anti-CD16/32	eBioscience	93	0.5 µg/10^6 cells	FACS
anti-Flk2	eBioscience	A2F10	1 µg/10^6 cells	FACS
anti-CD127	eBioscience	A7R34	1 µg/10^6 cells	FACS
anti-CD45	eBioscience	30-F11	0.5 µg/10^6 cells	FACS
Ki-67 Set	eBioscience	SolA15	0.25 µg/10^6 cells	FACS
anti-Suv39h1	Cell Signaling Technology	8729	1:500	Immunoblot
anti-GAPDH	Cell Signaling Technology	2118	1:1000	Immunoblot
anti-H3K9me3	Abcam	ab8898	1:1000	Immunoblot
anti-H3	Abcam	ab1791	1:50000	Immunoblot
anti-H3K9me3	Abcam	ab8898	2 µg/10^7 cells	ChIP
Isotype IgG	Abcam	ab171870	2 µg/10^7 cells	ChIP
Dynabeads protein G	Life technology	10004D	40 µl/test	ChIP
Dynabeads protein A	Life technology	10002D	40 µl/test	ChIP
Chaetocin	Sigma	C9492	Stork: 100 µM	5% DMSO in H_2O

Supplementary Table 2. qRT-PCR and ChIP-qPCR primers used in the study.

Gene	Gene ID	Forward primer sequence	Reverse primer sequence
SUV39H1	6839	CCTGCCCTCGGTATCTCTAAG	ATATCCACGCCATTTCACCAG
Suv39h1	20937	TGATTACAACATGCAAGTGGACC	CAGGCCCCTTCAGTTAGTCTG
Hoxb13	15408	TGCCCCTTGCTATAGGGAAT	ATTCTGGAAAGCAGCGTTTG
Six1	20471	ATGCTGCCGTCGTTTGGTT	CCTTGAGCACGCTCTCGTT
Mlf1	17349	ATGTTGCGCATACTTTCTCG	GATCACGATGTTCCGGATG
Pax5	18507	CAGAGTAGCTGCCCTGTCCA	AGGCCCACAGTCCTACCCTA
Dntt	21673	ACCCTGAGTTGTTCTCTGCC	AACTCGAAGAGCCTTCCTCA
Rn18s	19791	AGTCCCTGCCCTTTGTACACA	CGATCCGAGGGCCTCACTA
Actb	11461	TGAACCCTAAGGCCAACCGTGAAA	CAGGATGGCGTGAGGGAGAGCATAG
Hoxb13(for ChIP)	15408	AGCTCCTGCAATCTTGGGAC	GGCGTACAGGGGCTTTACTT

4. Supplemental References

1 Guo H, Chu Y, Wang L, Chen X, Chen Y, Cheng H *et al*. PBX3 is essential for leukemia stem cell maintenance in MLL-rearranged leukemia. Int J Cancer 2017; 141: 324-335.

2 Kim D, Pertea G, Trapnell C, Pimentel H, Kelley R, Salzberg SL. TopHat2: accurate alignment of transcriptomes in the presence of insertions, deletions and gene fusions. Genome biology 2013; 14: R36.

3 Trapnell C, Roberts A, Goff L, Pertea G, Kim D, Kelley DR *et al*. Differential gene and transcript expression analysis of RNA-seq experiments with TopHat and Cufflinks. Nat Protoc 2012; 7: 562-578.

4 Bernt KM, Zhu N, Sinha AU, Vempati S, Faber J, Krivtsov AV *et al*. MLL-rearranged leukemia is dependent on aberrant H3K79 methylation by DOT1L. Cancer cell 2011; 20: 66-78.
